# Restriction of HIV-1 infectivity by interferon and IFITM3 is counteracted by Nef

**DOI:** 10.1126/sciadv.adz7083

**Published:** 2025-10-01

**Authors:** Mahesh Agarwal, Kin Kui Lai, Isaiah Wilt, Saliha Majdoul, Abigail A. Jolley, Mary Lewinski, Alex A. Compton

**Affiliations:** ^1^ Center for Cancer Research, National Cancer Institute, Frederick, MD, USA.; ^2^ Department of Medicine, University of California San Diego, La Jolla, CA, USA.

## Abstract

The viral accessory protein Nef is a major determinant of HIV-1 pathogenicity in vivo. Nef is a multifunctional, immunomodulatory protein that downmodulates cell surface proteins, including CD4 and MHC class I (MHC-I), which are important for T cell–mediated immunity. In addition, Nef also regulates cell-intrinsic immunity—Nef boosts the infectivity of virions produced and released from HIV-infected cells, at least in part, by counteracting the antiviral activity of transmembrane proteins SERINC3 and SERINC5. Here, we show that Nef proteins derived from many primary isolates of HIV-1 restore infectivity in interferon-treated cells and confer resistance to the antiviral protein interferon-induced transmembrane protein 3 (IFITM3) in a SERINC3/5-independent manner. Using Nef derived from primary HIV-1 clade C infection, we found that Nef interacts with IFITM3 in membranes, reduces IFITM3 incorporation into HIV-1 virions, and restores HIV-1 fusion with target cells. Our findings reveal a previously unrecognized immunomodulatory role for Nef in the setting of the interferon-induced antiviral state during HIV-1 infection.

## Teaser

The viral accessory protein Nef promotes HIV-1 infectiousness by counteracting the antiviral properties of host protein IFITM3.

## INTRODUCTION

The viral accessory protein Nef is a major determinant of HIV-1 pathogenicity and is found in all members of the primate lentivirus family. Although nonessential for HIV-1 infection and replication in most tissue culture models, in vivo studies in humans and nonhuman primates showed that Nef contributes to disease progression (*1*, *2*). Nef is a myristoylated protein that associates with cellular membranes and regulates cellular signaling and vesicular trafficking pathways (*3*). By acting as a molecular bridge that links cellular cargo to the vesicular transport machinery, including the clathrin adaptors (AP-1, AP-2, and AP-3), Nef alters the abundance of a growing list of cell surface proteins (*4*, *5*). These include the immune-related CD4 and major histocompatibility complex (MHC) class I, which function in T cell signaling and antigen presentation, respectively (*6*–*8*).

Another well-characterized yet incompletely understood function of Nef is its ability to boost the infectivity of virions produced and released from HIV-infected cells. The Nef-mediated enhancement of viral infectivity has been attributed, at least in part, to counteraction of transmembrane serine incorporator (SERINC) family members SERINC3 and SERINC5, whose downmodulation and exclusion from HIV-1 virions are associated with enhanced virus fusion (*9*, *10*). The significance of HIV-1 restriction by SERINC3/5 and its antagonism by Nef is supported by the demonstration that the global spread of HIV-1 correlates with the potency of SERINC5 counteraction (*11*). Furthermore, Nef from primary isolates display elevated activity against SERINC as compared to Nef from the laboratory-adapted strain NL4-3 (*12*), and Nef isolated from HIV-1 controllers was shown to antagonize SERINC5 poorly compared to Nef isolated from HIV-1 progressors (*13*). The viral accessory proteins glycoGag and S2, encoded by murine leukemia virus (MLV) and equine infectious anemia virus, respectively, also exhibit SERINC3/5 counteraction activity despite a lack of homology with Nef (*9*, *14*, *15*). Collectively, these studies point to a protective role played by SERINC3/5 during host-retrovirus coevolution over time.

Nef antagonizes the functions of CD4 and MHC class I by sorting them into the endolysosome system. In the case of CD4, Nef uses a C-terminal di-leucine motif (in the context of ExxxLL) to form a complex with CD4 and AP-2, driving the internalization of CD4 into clathrin-coated pits and eventual delivery to the lysosomal compartment (*6*, *16*–*18*). It has been proposed that the same di-leucine motif of Nef enables AP-2–dependent internalization of SERINC3/5, indicating that a single-sequence determinant in Nef enables regulation of multiple host factors. It has been suggested that Nef may directly interact with SERINC3/5 to drive its internalization from the cell surface (*19*–*21*). However, the internalization of SERINC3/5 from the cell surface and exclusion from HIV-1 virions are not prerequisites for Nef-mediated counteraction, indicating that the mechanism by which Nef rescues infectivity remains poorly understood (*22*). Furthermore, it has been reported that HIV-1 Nef boosts infectivity of viruses produced in certain cell lines in an AP-2–dependent manner even in the absence of SERINC3/5 (*23*–*25*). Last, Nef variants from simian immunodeficiency viruses (SIVs) that fail to antagonize the restriction of HIV-1 by overexpressed human SERINC3/5 retain the ability to enhance HIV-1 infection and spread in human CD4^+^ T cells (*21*). These findings point to the existence of unidentified, Nef-sensitive antiviral proteins expressed in human cells that restrict HIV-1 infectivity.

We and others previously demonstrated that interferon-induced transmembrane (IFITM) proteins inhibit HIV-1 infectivity at the stage of fusion, with IFITM3 performing the most potent restriction (*26*–*28*). Similar to SERINC3/5, the anti-HIV activity of IFITM3 is associated with its incorporation into HIV-1 virions and restriction of virion fusion with target cells. However, unlike SERINC3/5, IFITM1 to IFITM3 are up-regulated by interferons (*29*). We recently showed that, in addition to HIV-1, IFITM3 also restricts the infectivity of MLV. Unexpectedly, we found that MLV deficient for glycoGag exhibited heightened sensitivity to restriction by IFITM3, while glycoGag overexpression reduced sensitivity of MLV to IFITM3 (*30*). Because MLV glycoGag and HIV-1 Nef were previously shown to share the ability to counteract SERINC3/5 and restore retrovirus infectivity, we hypothesized that Nef may also antagonize one or more human IFITM proteins. In further support of a functional relationship between Nef and IFITM proteins, it was recently reported that Nef reduced the levels of IFITM proteins in extracellular vesicles released from T cells, which was caused by downmodulation of IFITM from the cell surface (*31*). However, the regulation of IFITM proteins by Nef was not explored in the context of HIV-1 infection.

Here, we examined whether HIV-1 Nef proteins derived from diverse sources exhibit the ability to counteract the anti–HIV-1 activity of IFITM3. We identified primary isolates of Nef that counteracted the loss of HIV-1 infectivity resulting from type-I interferon treatment or IFITM3 overexpression, whereas Nef from laboratory-adapted molecular clones had little to no effect. Counteraction was apparently AP-2–dependent and correlated to the degree to which the C terminus of Nef interacted with IFITM3, as measured by co-immunoprecipitation and proximity ligation. Accordingly, IFITM3 counteraction by Nef was associated with reduced cell surface levels of IFITM3 and reduced IFITM3 incorporation into HIV-1 virions. Furthermore, we found that the Nef-IFITM3 interaction impaired IFITM3 homomultimerization and reduced the impact of IFITM3 on membrane fluidity, which were both previously shown by us to be important for the anti-HIV activity of IFITM3 (*32*). The antiviral activity of IFITM3 and its negation by Nef were unaffected by SERINC5 knockdown, suggesting that the counteraction of IFITM3 represents a unique function of Nef. By demonstrating that HIV-1 Nef targets an interferon-stimulated gene product, we reveal a previously unrecognized role for HIV-1 Nef in promoting virus infectivity in the setting of the interferon-induced antiviral state.

## RESULTS

### Nef from some primary isolates of HIV-1 counteract restriction by IFITM3 and type-I interferon

**Fig. 1.**
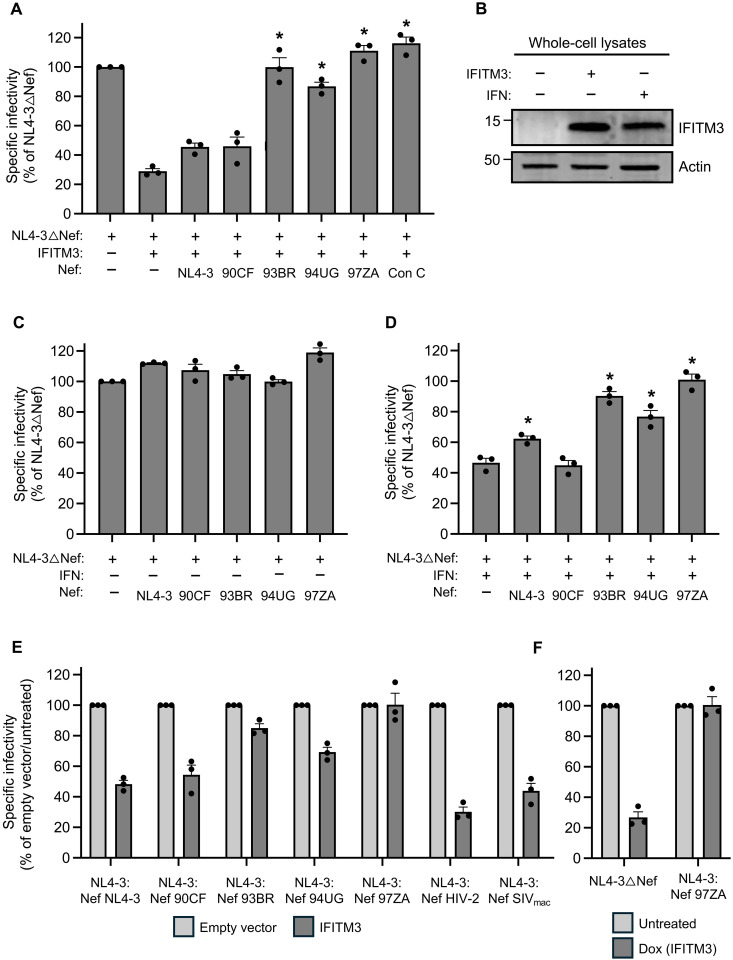
Nef counteracts restriction of HIV-1 infectivity by type-I interferon and IFITM3. (**A**) HEK293T were cotransfected with NL4-3△Nef (2.0 μg), pCMV-IFITM3/empty vector (0.5 μg), and pBJ-Nef-HA (0.25 μg). Virus harvested 24 hours posttransfection and quantified by p24 ELISA. A 25 ng of p24 equivalent added to TZM-bl. Infection scored by anti-Gag immunostaining. Infectivity shown as mean and SD (normalized to NL4-3△Nef alone, set to 100%). Differences statistically significant from NL4-3△Nef + IFITM3 by one-way analysis of variance (ANOVA) (*P < 0.05). (**B**) HEK293T cells were transfected with pCMV-IFITM3/empty vector (0.5 μg) or treated with type-I interferon (IFN) (~30 units per well), and whole-cell lysates were prepared. SDS–polyacrylamide gel electrophoresis (SDS-PAGE) and immunoblotting performed with anti-IFITM3 and anti-actin. Numbers/tick marks indicate position and size (kilodaltons) of ladder standard. (**C**) HEK293T were cotransfected with NL4-3△Nef (2.0 μg) and pBJ-Nef-HA (0.25 μg). Infectivity shown as mean and SD (normalized to NL4-3△Nef alone, set to 100%). (**D**) As in (C), but transfected cells treated with type-I interferon overnight before virus harvest. Infectivity shown as mean and SD [normalized to NL4-3△Nef in (C), set to 100%]. Differences statistically significant from NL4-3△Nef + interferon by one-way ANOVA (*P < 0.05). (**E**) HEK293T were cotransfected with NL4-3 encoding Nef in cis (2.0 μg) and pCMV-IFITM3/empty vector (0.5 μg). Infectivity shown as mean and SD (normalized to NL4-3△Nef + empty vector, set to 100%). (**F**) SupT1 Tet-ON IFITM3 were infected with NL4-3△Nef or NL4-3 encoding 97ZA Nef for 48 hours, washed, and treated with doxycycline (500 ng/ml) for 18 hours. Virus was harvested and quantified by p24 ELISA. A 100 ng of p24 equivalents was added to TZM-bl. Infection scored by anti-Gag. Virus infectivity shown as mean and SD [normalized to untreated (no doxycycline), set to 100%]. Filled circles represent independent transfections. Con, consensus; Dox, doxycycline.

To measure the antiviral activity of IFITM3 in HIV-1 producer cells and its sensitivity to Nef, we produced Nef-deficient HIV-1 from human embryonic kidney (HEK) 293T cells transfected with human IFITM3 (untagged) and individual Nef proteins from diverse sources [tagged with hemagglutinin (HA)]. Virus produced was quantified by p24 Gag enzyme-linked immunosorbent assay (ELISA), and infectivity was measured on TZM-bl reporter cells inoculated with equal quantities of virus. The overexpression of IFITM3 reduced Nef-deficient HIV-1 by approximately fivefold, but coexpression of IFITM3 with Nef resulted in partial or complete recovery of infectivity depending on the Nef isolate (Fig. 1A and fig. S1A). IFITM3 protein levels following transient transfection of HEK293T were similar to those induced by type-I interferon treatment of the same cell line (Fig. 1B). The expression of Nef from the laboratory-adapted molecular clone NL4-3 or a Nef isolate obtained during acute clade H infection (90CF056) (*33*) resulted in modestly improved infectivity. In contrast, Nef derived from other acute primary isolates of HIV-1 (93BR020; clade F and 94UG114; clade D) (*33*) restored HIV-1 infectivity to a greater extent, while a Nef isolate from clade C (97ZA013) fully restored HIV-1 infectivity (Fig. 1A). We found that 97ZA013 Nef did not significantly boost Nef-deficient HIV-1 infectivity in the absence of IFITM3, while the restoration of infectivity in the presence of IFITM3 was a function unique to Nef but not Vpu in the 97ZA013 strain (fig. S1B). 97ZA013 Nef also counteracted restriction of MLV by IFITM3 to a similar extent as glycoGag (fig. S1C). Thus, some HIV-1 Nef proteins exhibit the capacity to counteract the restriction of retrovirus infectivity by IFITM3. Since we and others previously demonstrated that the paralog of IFITM3, IFITM2, also inhibits HIV-1 infectivity (albeit to a lesser extent than IFITM3) (*26*, *28*, *34*), we assessed if 97ZA013 Nef regulated the antiviral impacts of IFITM2 expression in virus-producing cells. Similar to IFITM3, the restriction of HIV-1 infectivity by IFITM2 was also counteracted by 97ZA013 Nef (fig. S1, D and E). To determine whether the complete counteraction of IFITM3 by Nef is a conserved feature during early clade C infections, we synthesized a consensus Nef sequence from primary isolates obtained from 105 individuals acutely infected with HIV-1 clade C (*35*). Similarly to the 97ZA013 isolate, the clade C consensus Nef also fully counteracted the antiviral activity of IFITM3 (Fig. 1A). Thus, Nef from primary HIV-1 isolates can antagonize human IFITM3, and this is particularly evident for Nef originating from acute clade C, which is the most prevalent form of HIV-1 and accounts for ~50% of HIV-1 infections globally (*36*).

Since IFITM3 is an interferon-stimulated gene, we next examined whether Nef boosted the infectivity of HIV-1 produced from HEK293T cells treated with type-I interferon. Interferon treatment increased the abundance of IFITM3 protein, but no such increase was observed for IFITM2 using a specific anti-IFITM2 antibody (fig. S1, E and F) (*37*). In the absence of interferon treatment, the expression of different Nef constructs had little to no effect on the infectivity of Nef-deficient HIV-1 (Fig. 1C), suggesting that these Nef proteins do not boost infectivity by counteracting constitutively expressed antiviral factors in HEK293T. However, in cells treated with type-I interferon, certain Nef proteins significantly elevated virus infectivity. Specifically, the three Nef proteins obtained from primary isolates that significantly restored infectivity in cells overexpressing IFITM3 (93BR020, 94UG114, and 97ZA013) also restored infectivity in interferon-treated cells (Fig. 1D). These data suggest that Nef proteins from certain primary isolates affect the susceptibility of HIV-1 to restriction by interferon-induced antiviral factors including IFITM3.

To confirm our findings on the counteractive role played by certain Nef proteins, we also assessed Nef-mediated counteraction of overexpressed IFITM3 using recombinant, full-length HIV-1 encoding different Nef proteins in cis. As was observed when transfecting Nef in trans to rescue a Nef-deficient HIV-1, we found that HIV-1 encoding Nef from certain primary isolates (93BR020, 94UG114, and 97ZA013) increased the infectivity of virus produced in the presence of IFITM3, while virus with NL4-3 Nef and 90CF056 Nef did so to a lesser extent (Fig. 1E). We also found that SIVmac Nef did little to counteract restriction by IFITM3, and HIV-2 Nef was completely inactive in this regard. These data indicate that Nef proteins from primary isolates of HIV-1 can antagonize IFITM3 either when overexpressed or naturally expressed in the context of the provirus.

To measure whether Nef confers resistance to IFITM3 when virus is produced from natural target cells of HIV-1, we measured the infectivity of HIV-1 produced from SupT1 lymphocytes stably expressing IFITM3 under the control of doxycycline (SupT1-IFITM3). In these experiments, SupT1-IFITM3 cells were infected with Nef-deficient HIV-1 or recombinant HIV-1 encoding 97ZA013 Nef, and IFITM3 expression was induced or not with doxycycline post-inoculation. Viral supernatants were then collected, and equivalent virus inputs were tested for infectivity in TZM-bl cells. Our results showed that the infectivity of Nef-deficient HIV-1 produced from SupT1-IFITM3 was reduced fourfold upon IFITM3 induction, while virus encoding 97ZA013 Nef was unaffected by IFITM3 induction (Fig. 1F). Notably, the amount of ectopic IFITM3 protein induced by doxycycline was similar to the amount of endogenous IFITM3 induced by type-I interferon treatment of SupT1 cells (fig. S1F). These findings demonstrate that the antiviral activity of IFITM3 in HIV-producing cells, including T cells, and its resultant impact on virus infectivity are counteracted by certain Nef proteins derived from primary isolates of HIV-1.

### Nef counteracts IFITM3 by interacting with it, excluding it from HIV-1 virions, and restoring HIV-1 fusion with target cells

**Fig. 2.**
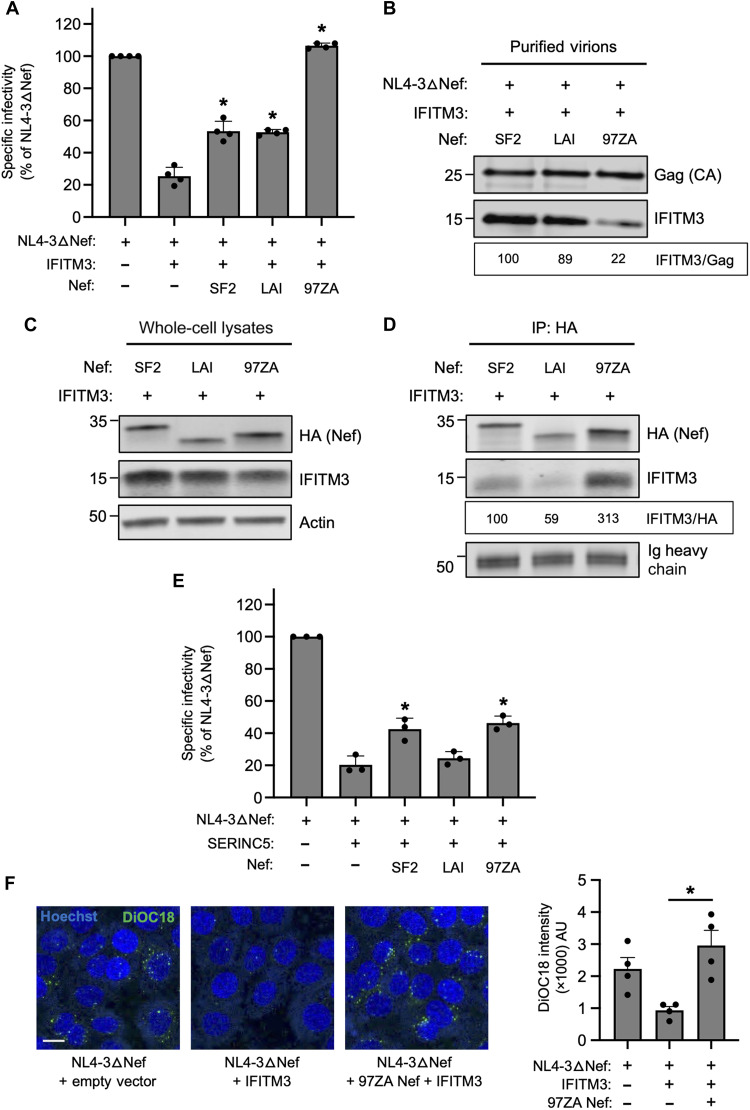
Nef restores HIV-1 entry by interacting with IFITM3 and partially excluding it from virions. (**A**) HEK293T cells were cotransfected with NL4-3△Nef (2.0 μg), pCMV-IFITM3/empty vector (0.5 μg), and pBJ-Nef-HA (0.25 μg). Produced virus harvested 24 hours posttransfection and quantified by p24 ELISA. A 25 ng of p24 equivalents added to TZM-bl. Infection scored by anti-Gag immunostaining. Infectivity shown as mean and SD (normalized to NL4-3△Nef alone, set to 100%). Differences statistically significant from NL4-3△Nef + IFITM3 by one-way ANOVA (*P < 0.05). (**B**) Cells were transfected as in (A) and virus harvested at 24 hours posttransfection and purified by ultracentrifugation over a 20% sucrose cushion at 25,000 rpm for 1 hour, and viral pellets were subjected to SDS-PAGE and immunoblotting with anti-Gag and anti-IFITM3. (**C**) HEK293T cells were cotransfected with pCMV-IFITM3/empty vector (0.50 μg) and pBJ-Nef-HA (0.25 μg), and whole-cell lysates were subjected to SDS-PAGE and immunoblotting. (**D**) From whole-cell lysates in (C), Nef immunoprecipitated with anti-HA followed by SDS-PAGE and immunoblotting. Ig heavy chain is loading control. Co-immunoprecipitations were performed twice from independent transfections. Mean IFITM3/HA ratio calculated for indicated lanes (normalized to SF2 Nef + IFITM3, set to 100%). (**E**) As in (A) but with pBJ-SERINC5/empty vector (0.1 μg). (**F**) As in (A) but produced virus was harvested 24 hours posttransfection and labeled with SP-DiOC18. A 40 ng of p24 equivalents of labeled virus was added to TZM-bl on ice for 1 hour, and then cells were incubated at 37°C for 1 hour and subsequently fixed. DiOC18 fluorescence intensity shown as means and SD. Filled circles represent fields of view containing 8 to 15 cells each from two experiments. Scale bar, 10 μm. Differences statistically significant between indicated conditions by one-way ANOVA (*P < 0.05). IP, immunoprecipitation; CA, capsid; AU, arbitrary units.

To gain insight into the mechanistic basis for Nef-mediated antagonism of IFITM3 and the basis for antagonism encoded by Nef obtained from primary virus isolates, we compared the effects of two Nef proteins obtained from molecular clones of HIV-1 (SF2 and LAI) and compared them to the activity of 97ZA013 Nef obtained from primary clade C HIV-1 (hereafter referred to as 97ZA). As was observed for NL4-3 Nef (Fig. 1A), SF2 Nef and LAI Nef modestly boosted HIV-1 infectivity in the presence of IFITM3, while 97ZA Nef fully counteracted IFITM3 (Fig. 2A). To understand why 97ZA Nef counteracted IFITM3 to a much greater extent than SF2 Nef and LAI Nef, we assessed the degree to which IFITM3 incorporated into HIV-1 virions in their presence. We previously found that restriction of HIV-1 infectivity is associated with the cell surface expression and virion incorporation of IFITM3 oligomers (*26*, *27*, *32*). Consistent with its unique capacity to restore infectivity in the presence of IFITM3, the amount of virion-associated IFITM3 protein was reduced by 97ZA Nef (Fig. 2B). We confirmed that 97ZA Nef wild type (WT) reduced the amount of IFITM3 incorporated into HIV-1 virions relative to 97ZA Nef G2A, a loss-of-function mutant that served as a negative control (fig. S2A). While 97ZA Nef reduced IFITM3 levels in purified virions, it did not reduce the amount of IFITM3 detected in extracellular vesicles present in cell culture medium (fig. S2B).

We next tested whether Nef and IFITM3 have the potential to interact following transient transfection in HEK293T cells. In transfected whole-cell lysates, we observed that total IFITM3 protein detected by immunoblotting was only modestly affected by the different Nef proteins, with 97ZA Nef slightly decreasing IFITM3 protein (Fig. 2C). Gel migration differences reflect natural variation in sequence and structure among Nef proteins. When Nef was immunoprecipitated from lysates, we observed that pull-down of IFITM3 occurred, and 97ZA Nef co-immunoprecipitated with IFITM3 to a greater extent than the other Nefs (Fig. 2D). Therefore, these data suggest that IFITM3 counteraction by Nef involves a direct or indirect interaction between IFITM3 and Nef, which is associated with partial virion exclusion of IFITM3. We tested the same Nef proteins for counteraction of SERINC5-mediated restriction of HIV-1 infectivity and found that the impact of Nef did not follow the same pattern as was observed with IFITM3. Specifically, SF2 Nef and 97ZA Nef partially counteracted SERINC5, and they did so to a similar magnitude (Fig. 2E). Thus, the molecular determinants by which Nef counteracts SERINC5 are not identical to those governing counteraction of IFITM3. We confirmed that neither the antiviral activity of IFITM3 nor the Nef-mediated boost to infectivity in the presence of IFITM3 was dependent on SERINC5 by performing experiments in SERINC5 knockdown cells (fig. S2, C and D). These findings indicate that antagonism of IFITM3 by Nef from primary isolates is a unique and separate function from that of SERINC5 antagonism.

Because we previously demonstrated that IFITM3 reduces HIV-1 entry at the level of membrane fusion with target cells (*26*), we tested whether the impact of 97ZA Nef on HIV-1 infectivity occurs at the step of virus fusion. Nef-deficient virus was produced, in the absence or presence of IFITM3, and labeled with a self-quenching concentration of DiOC18. Labeled virus was added to TZM-bl cells, and DiOC18 dequenching (fluorescence) was measured in living cells. Fluorescence reported membrane fusion between labeled virus and unlabeled cellular membranes, since it was diminished in the presence of the fusion inhibitor T-20 (fig. S2E). In this assay, the expression of IFITM3 in virus-producing cells resulted in significantly reduced virus fusion, while fusion was restored upon expression of 97ZA Nef (Fig. 2F). These findings suggest that Nef counteracts IFITM3 by interacting with it, partially excluding it from HIV-1 virions, and restoring fusogenic potential to virions.

### Nef uses a ExxxLL motif to counteract IFITM3 and downmodulates IFITM3 from the cell surface

**Fig. 3.**
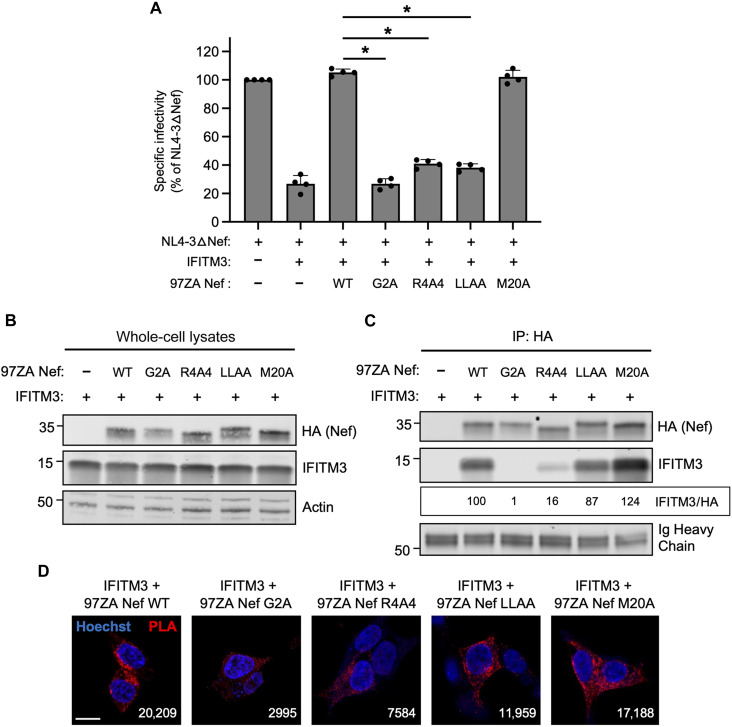
Nef-mediated counteraction of IFITM3 requires a di-leucine motif known to recruit the endocytic adaptor AP-2. (**A**) HEK293T cells were cotransfected with NL4-3△Nef (2.0 μg), pCMV-IFITM3 or empty vector (0.5 μg), and pBJ-97ZA Nef-HA encoding WT or mutant (0.25 μg). Produced virus was harvested 24 hours posttransfection and quantified by p24 ELISA. A 25 ng of p24 equivalents was added to TZM-bl, and infection was scored by anti-Gag immunostaining. Infectivity is shown as mean and SD (normalized relative to NL4-3△Nef alone, which was set to 100%). Filled circles represent biological replicates (independent transfections). Differences between the indicated conditions that were statistically significant by one-way ANOVA are indicated by (*) (*P* < 0.05). (**B**) HEK293T cells were cotransfected with pCMV-IFITM3 or empty vector (0.50 μg) and pBJ-97ZANef-HA encoding WT or mutant (0.25 μg), and whole-cell lysates were subjected to SDS-PAGE and immunoblotting with anti-HA, anti-IFITM3, and anti-actin. (**C**) From whole-cell lysates in (B), Nef proteins were immunoprecipitated with anti-HA followed by SDS-PAGE and immunoblotting with anti-HA and anti-IFITM3. Ig heavy chain served as loading control. Co-immunoprecipitations were performed twice from independent transfections. The mean IFITM3/HA ratio was calculated for the indicated lanes (normalized to 97ZA Nef WT + IFITM3, set to 100%). (**D**) HEK293T cells were transfected as in (B) and fixed, and proximity ligation assay was performed using anti-IFITM3 and anti-HA followed by confocal microscopy. Nuclei were stained with Hoechst. Scale bar, 10 μm. White numbers indicate the mean PLA signal intensities as measured by corrected total cell fluorescence calculated from 6 to 12 cells per condition.

**Fig. 4.**
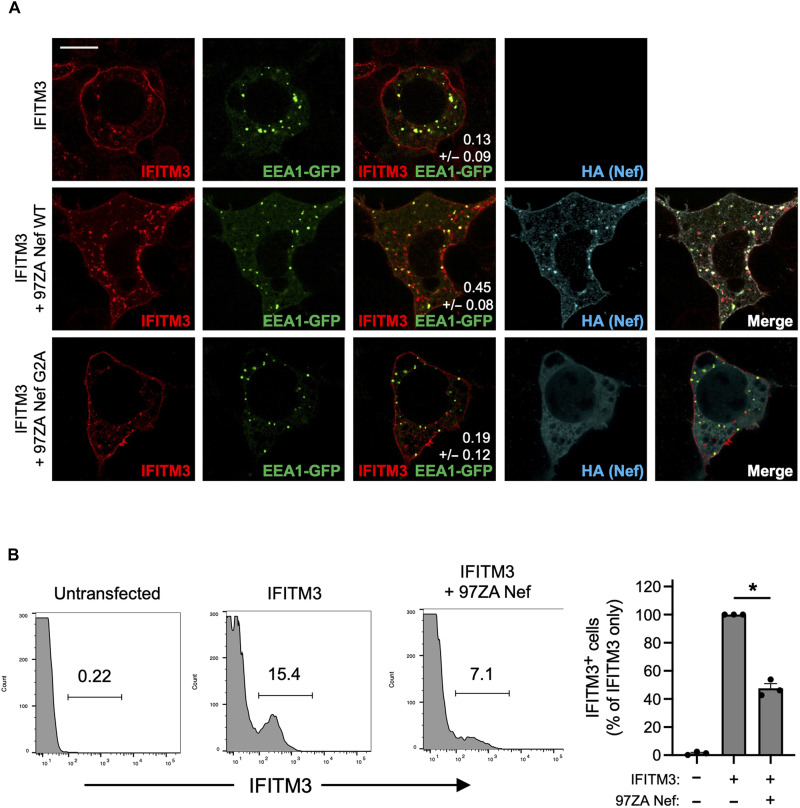
Nef downmodulates IFITM3 from the cell surface and promotes its accumulation in early endosomes. (**A**) HEK293T cells were cotransfected with pCMV-IFITM3 (0.50 μg), pBJ-97ZA Nef-HA encoding WT or G2A mutant (0.25 μg), and EEA1-GFP (0.50 μg), fixed, immunostained with anti-IFITM3 and anti-HA, and examined by immunofluorescence confocal microscopy. Pearson’s correlation coefficients were calculated between IFITM3 and EEA1-GFP in 20 cells per condition and presented as mean and SD (white numbers in middle column). (**B**) HEK293T cells were transfected with pCMV-IFITM3 (0.50 μg) alone or both pCMV-IFITM3 and pBJ-97ZANef-HA (0.25 μg), and living, intact cells were stained with anti-IFITM3. Subsequently, cells were fixed, and IFITM3-positive cells were quantified by flow cytometry. A representative example of dot plot histograms is shown on the left, and the summary data of three biological replicates (independent transfections) are shown on the right. IFITM3-positive cells in each condition are shown as means and SD (normalized to cells transfected with IFITM3 alone, set to 100%). Differences between the indicated conditions that were statistically significant by Student’s *t* test are indicated by (*) (*P* < 0.05). Scale bar, 15 μm.

To learn more about the mechanisms employed by Nef to counteract IFITM3, we introduced mutations into 97ZA Nef that have been previously characterized to disrupt various functions of Nef. Compared to 97ZA Nef WT, which fully restored HIV-1 infectivity in the presence of IFITM3, the mutation of the myristoylation site of Nef (G2A) resulted in total loss of IFITM3 counteraction (Fig. 3A). Similarly, mutation of four basic residues (R17/19/21/22A or “R4A4”) in the amino terminus of Nef also prevented counteraction of IFITM3. The G2A and R4A4 mutations are known to disrupt Nef localization to cellular membranes (*38*–*40*), indicating that membrane association is an important part of how Nef counteracts IFITM3. To identify the cellular machinery that Nef coopts to counteract IFITM3, we introduced L165/166A (“LLAA”) to disrupt the ExxxLL dileucine motif found in the C-terminal loop of Nef, which was previously shown to enable AP-2–dependent downmodulation of CD4 (*41*–*43*). We also tested the M20A mutation previously shown to disrupt AP-1–mediated downmodulation of MHC class I (*7*, *44*). The LLAA mutations in Nef resulted in near-complete loss of IFITM3 counteraction, while M20A had no effect (Fig. 3A). These results indicate that the mechanism used by 97ZA Nef to counteract IFITM3 resembles the mechanism used by Nef to counteract CD4, while it is distinct from the mechanism used to counteract MHC class I. Co-immunoprecipitation experiments revealed that the G2A and R4A4 mutations in Nef inhibited the interaction with IFITM3, while the LLAA and M20A did not (Fig. 3, B and C). Since Nef LLAA exhibits decreased capacity to counteract IFITM3 (Fig. 3A), yet it still interacts with IFITM3 (Fig. 3C), the ExxxLL motif likely contributes to IFITM3 counteraction at a step downstream of IFITM3 binding, such as AP-2 recruitment. We also used the proximity ligation assay (PLA) to confirm that 97ZA Nef WT and IFITM3 interact in intact cells, and with this approach, we confirmed that the interaction is disrupted by G2A and R4A4 in Nef (Fig. 3D). These results suggest that 97ZA Nef counteracts the antiviral activity of IFITM3 and restores virus infectivity by interacting with IFITM3 in membranes and by recruiting AP-2 via the ExxxLL dileucine motif. Because we previously showed that MLV glycoGag counteracted IFITM3, while a glycoGag mutant deficient for AP-2 binding (Y36A) did not (*30*), these results suggest that glycoGag and Nef use a similar mechanism to counteract IFITM3. To extend those findings and test whether Nef affects the subcellular localization of IFITM3, we cotransfected HEK293T with IFITM3 and either 97ZA Nef WT or G2A and performed confocal immunofluorescence microscopy. In the absence of Nef, IFITM3 could be detected at the cell surface and intracellular compartments including early endosomes [identified by EEA1–green fluorescent protein (GFP)], as demonstrated previously (*26*, *27*, *32*) (Fig. 4A). However, in the presence of 97ZA Nef WT, there was an enrichment of IFITM3 in EEA1-GFP^+^ puncta, and a portion of Nef protein was found to colocalize with IFITM3 and EEA1-GFP. Under these experimental conditions, we confirmed that Nef interacted with IFITM3, as measured by PLA (fig. S3A) and co-immunoprecipitation (fig. S3B), while little to no interaction between Nef and EEA1-GFP was detected (fig. S3, A and B). Furthermore, there was an apparent reduction of IFITM3 from the cell surface in the presence of 97ZA Nef WT. In contrast, the expression of 97ZA Nef G2A, which localized diffusely throughout the cytosol, did not affect the localization of IFITM3 (Fig. 4A). We quantified the amount of IFITM3 protein on the cell surface by immunostaining living, transfected cells with an anti-IFITM3 antibody, followed by fixation and analysis by flow cytometry. We found that 97ZA Nef WT reduced IFITM3 at the cell surface by ~60% (Fig. 4B and fig. S3C). These results are consistent with a mechanistic model whereby Nef interacts with IFITM3 and uses AP-2 to redirect IFITM3 from the plasma membrane to early endosomes, resulting in decreased virion incorporation of IFITM3 and reduced inhibition of HIV-1 infectivity.

### Nef impairs IFITM3 oligomerization and restores membrane fluidity in IFITM3-expressing cells

**Fig. 5.**
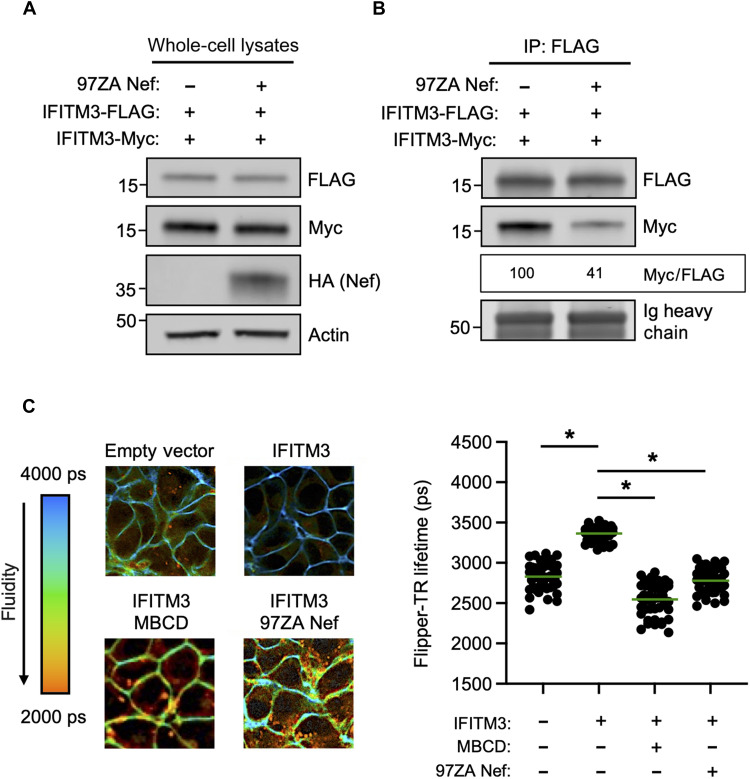
Nef interferes with IFITM3 oligomerization and restores membrane fluidity in IFITM3-expressing cells. (**A**) HEK293T cells were cotransfected with pCMV-IFITM3-FLAG (0.50 μg) and pCMV-IFITM3-Myc (0.50 μg) alone or in combination with pBJ-97ZANef-HA (0.25 μg), and whole-cell lysates were subjected to SDS-PAGE and immunoblotting with anti-FLAG, anti-Myc, anti-HA, and anti-actin. (**B**) From whole-cell lysates in (A), IFITM3-FLAG was immunoprecipitated with anti-FLAG antibody, followed by SDS-PAGE and immunoblotting with anti-FLAG and anti-Myc. Ig heavy chain served as loading control. Co-immunoprecipitations were performed twice from independent transfections. The mean Myc/FLAG ratio was calculated for the indicated lanes (normalized to IFITM3-FLAG and IFITM3-Myc, set to 100%). (**C**) HEK293T stably expressing IFITM3 or empty vector was untransfected or transfected with pBJ-97ZA Nef-HA (0.25 μg). In the indicated condition, cells were pretreated with 5 mM MBCD for 2 hours. All conditions were then incubated with the membrane order probe Flipper-TR at a final concentration of 1 μM for 10 min. Membrane fluidity of individual cells was measured by fluorescence lifetime imaging. Left: Representative images of fluorescence lifetimes, with blue indicating long lifetimes (less fluid, more rigid) and red indicating short lifetimes (more fluid, less rigid). Right: Lifetimes were plotted, mean lifetimes are indicated by green lines, and differences between the indicated conditions statistically significant by one-way ANOVA are indicated by (*) (*P* < 0.05). Filled circles correspond to lifetime measurements of individuals cells (50 cells per condition). ps, picoseconds; MBCD, methyl-beta-cyclodextrin.

In addition to partially reducing IFITM3 protein quantity present within virus particles, it remained a possibility that Nef influenced IFITM3 function through additional means. We previously published that oligomerization of IFITM3 is important for its known antiviral functions, including the inhibition of HIV-1 infectivity (*32*). To measure the degree to which IFITM3 forms oligomers in the absence and presence of Nef, we cotransfected HEK293T with FLAG-tagged IFITM3 and Myc-tagged IFITM3. The pull down of Myc-IFITM3 with immunoprecipitated FLAG-IFITM3 demonstrated that these constructs form multimers in transfected cells, as demonstrated previously (*32*) (Fig. 5A). However, the expression of 97ZA Nef resulted in reduced co-immunoprecipitation of FLAG-IFITM3 and Myc-IFITM3, suggesting that Nef interferes with IFITM3 oligomerization (Fig. 5B). We and others previously reported that IFITM3 reduces cellular membrane fluidity (*32*, *45*). Furthermore, an oligomerization-defective mutant of IFITM3 exhibited reduced impact on membrane fluidity and reduced impact on HIV-1 infectivity (*32*). Therefore, we measured whether Nef regulated membrane fluidity in cells expressing IFITM3. By measuring membrane fluidity in living cells using fluorescence lifetime imaging of the membrane order probe Flipper-TR (*46*), we observed that IFITM3 reduced membrane fluidity as expected, as demonstrated by increased Flipper-TR lifetimes (Fig. 5C). The reduced fluidity observed in IFITM3-expressing cells was reversed using the cholesterol extracting agent methyl-beta-cyclodextrin (MBCD). Notably, 97ZA Nef expression had a similar effect, restoring membrane fluidity to normal levels in IFITM3-expressing cells (Fig. 5C). These findings suggest that 97ZA Nef not only modifies the subcellular localization of IFITM3 but also functionally inactivates IFITM3 by inhibiting IFITM3 oligomerization and by inhibiting the effect of IFITM3 oligomers on membrane fluidity.

### Interaction with and counteraction of IFITM3 is governed by the C terminus of Nef

**Fig. 6.**
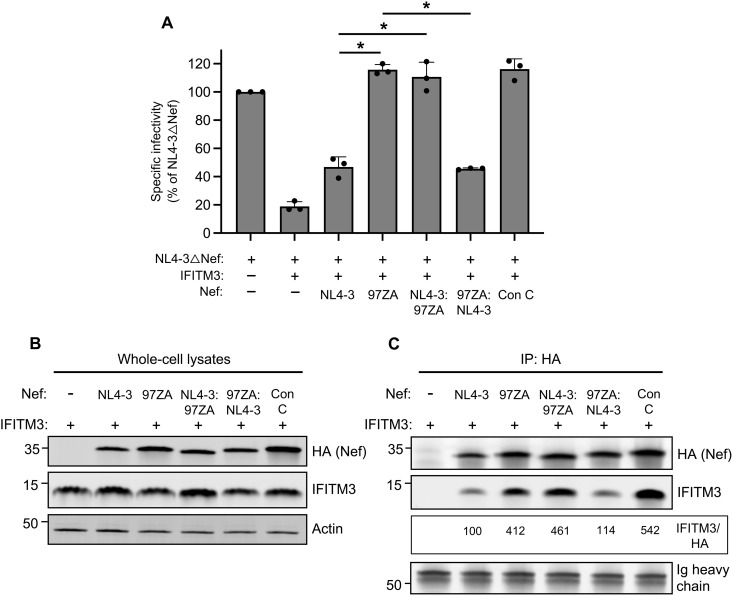
The C terminus of Nef contains determinants for interacting with and counteracting IFITM3. (**A**) HEK293T were cotransfected with NL4-3△Nef (2.0 μg); pCMV-IFITM3 or empty vector (0.5 μg); and pBJ-NL4-3 Nef-HA, pBJ-97ZA Nef-HA, or chimeric Nef, whereby the C-termini of NL4-3 and 97ZA Nef were swapped (0.25 μg). Produced virus was harvested 24 hours posttransfection and quantified by viral p24 ELISA. A 25 ng of p24 equivalent of fresh virus-containing supernatants was added to TZM-bl, and infection was scored by anti-Gag immunostaining. Infectivity is shown as mean and SD (normalized relative to NL4-3△Nef alone, which was set to 100%). Filled circles represent biological replicates (independent transfections). Differences between the indicated conditions statistically significant by one-way ANOVA are indicated by (*) (*P* < 0.05). (**B**) HEK293T cells were cotransfected with pCMV-IFITM3 (0.50 μg) and pBJ-NL4-3 Nef-HA, pBJ-97ZA Nef-HA, or chimeric Nef, whereby the C-termini of NL4-3 and 97ZA Nef proteins were swapped (0.25 μg), and whole-cell lysates were subjected to SDS-PAGE and immunoblotting with anti-HA, anti-IFITM3, and anti-actin. (**C**) From whole-cell lysates in (B), Nef proteins were immunoprecipitated with anti-HA and subjected to SDS-PAGE and immunoblotting with anti-HA and anti-IFITM3 (Ig heavy chain served as loading control). Co-immunoprecipitations were performed twice from independent transfections. The mean IFITM3/HA ratio was calculated for the indicated lanes (normalized to NL4-3 Nef and IFITM3, set to 100%). Con, consensus.

The counteraction through inactivation of IFITM3 by Nef may be a consequence of Nef interacting with IFITM3, either directly or indirectly, in membranes. Therefore, we sought to understand the determinants for IFITM3 binding, and its relationship to IFITM3 counteraction, by producing chimeric Nef proteins. Since NL4-3 Nef and 97ZA Nef differentially counteract IFITM3 (Fig. 1A), we swapped the C-termini of these Nef proteins and tested them for their ability to rescue Nef-deficient HIV-1 infectivity in the presence of IFITM3. While NL4-3 Nef only modestly counteracted restriction by IFITM3, 97ZA Nef did so fully (Fig. 6A). However, when the C terminus of NL4-3 Nef was replaced by that of 97ZA Nef, a complete restoration of HIV-1 infectivity was observed (Fig. 6A). The reverse swap, whereby the C terminus of 97ZA Nef was replaced with that of NL4-3 Nef, resulted in a loss of IFITM3 counteraction. These results demonstrate that the C terminus of Nef contains determinants that dictate IFITM3 counteraction. Immunoprecipitation experiments revealed that IFITM3 pulled down to a greater extent with 97ZA Nef relative to NL4-3 Nef, consistent with the greater capacity for 97ZA Nef to counteract IFITM3 functionally (Fig. 6, B and C). Meanwhile, the quantity of IFITM3 pulled down by Nef chimeras indicated that the C terminus of Nef also governs the interaction with IFITM3 (Fig. 6, B and C). These results suggest that Nef-mediated counteraction of IFITM3 and restoration of HIV-1 infectivity are functionally linked to a physical interaction between IFITM3 and Nef, one that is governed by determinants in the C terminus of Nef.

**Fig. 7.**
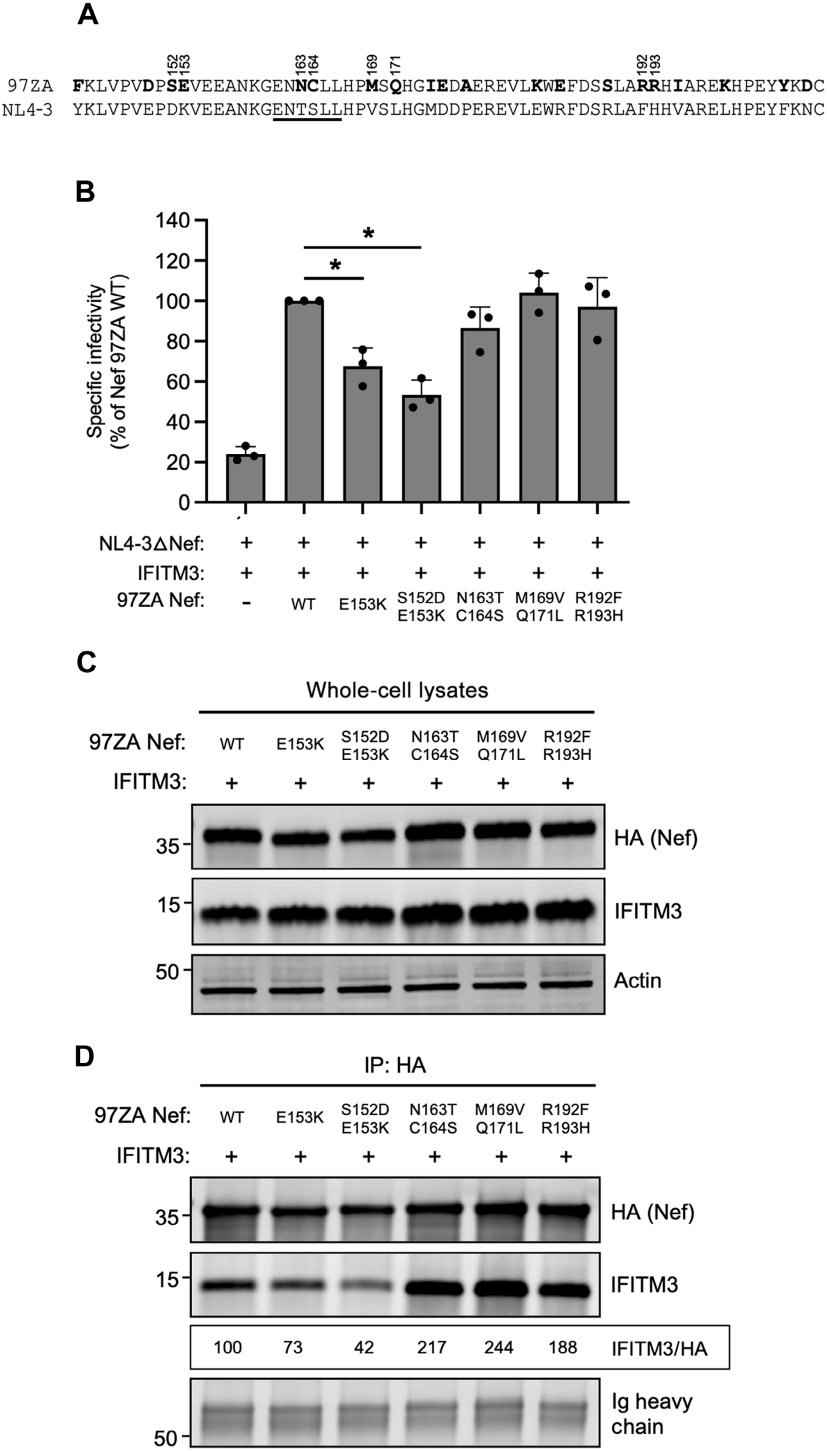
Residues 152 and 153 in the C terminus of Nef are determinants for interacting with and counteracting IFITM3. (**A**) Protein sequence alignment of the C-termini (residues 143 to 206) of 97ZA Nef and NL4-3 Nef. Amino acid residues unique to 97ZA Nef are indicated by bold lettering. The ExxxLL motif previously described to coordinate AP-2 dependent downmodulation of CD4 is underlined. (**B**) HEK293T cells were cotransfected with NL4-3△Nef (2.0 μg), pCMV-IFITM3 or empty vector (0.5 μg), and pBJ-97ZA Nef-HA WT or the indicated mutant (0.25 μg). Produced virus was harvested 24 hours posttransfection and quantified by p24 ELISA. A 25 ng of p24 equivalents of virus was added to TZM-bl, and infection was scored by anti-Gag immunostaining. Infectivity is shown as mean and SD (normalized relative to NL4-3△Nef + IFITM3 + 97ZA Nef WT, set to 100%). Filled circles represent biological replicates (independent transfections). Differences between the indicated conditions statistically significant by one-way ANOVA are indicated by (*) (*P* < 0.05). (**C**) HEK293T cells were cotransfected with pCMV-IFITM3 (0.50 μg) and pBJ-97ZA Nef-HA WT or mutant (0.25 μg), and whole-cell lysates were subjected to SDS-PAGE and immunoblotting with anti-HA, anti-IFITM3, and anti-actin. (**D**) From whole-cell lysates in (C), Nef proteins were immunoprecipitated with anti-HA and subjected to SDS-PAGE and immunoblotting with anti-HA and anti-IFITM3 (Ig heavy chain served as a loading control). Co-immunoprecipitations were performed twice from independent transfections. The mean IFITM3/HA ratio was calculated for the indicated lanes (normalized to 97ZA Nef and IFITM3, set to 100%).

There exist 20 amino acid differences between the C-termini of NL4-3 Nef and 97ZA Nef (Fig. 7A). We swapped amino acid residues, individually or in pairs, into 97ZA Nef to assess whether they negatively impacted the counteraction of IFITM3 and its interaction with IFITM3. We found that E153K caused a significant reduction in IFITM3 counteraction by 97ZA Nef, and this was reduced further when combined with S152D (Fig. 7B). In contrast, the N163T + C164S mutations only somewhat reduced IFITM3 counteraction potential despite their location in the ExxxLL motif (Fig. 7B). The others tested (M169V + Q171L and R192F + R193H) did not significantly affect IFITM3 counteraction by 97ZA Nef. The mutations that significantly reduced IFITM3 counteraction (S152D + E153K) also significantly impaired IFITM3 pull-down (Fig. 7, C and D). In contrast, mutations at residues 163, 164, 169, 171, 192, and 193 did not impair, but rather slightly enhanced, the pull-down of IFITM3. Therefore, S152 and E153 in 97ZA Nef are key contributors to the enhanced capacity for this Nef to counteract IFITM3. Furthermore, these results pinpoint key residues in Nef that control binding with IFITM3 and further demonstrate that the interaction between Nef and IFITM3 is pivotal to the counteraction of IFITM3 antiviral activity.

### Nef from clade B T/F HIV-1 counteracts IFITM3

**Fig. 8.**
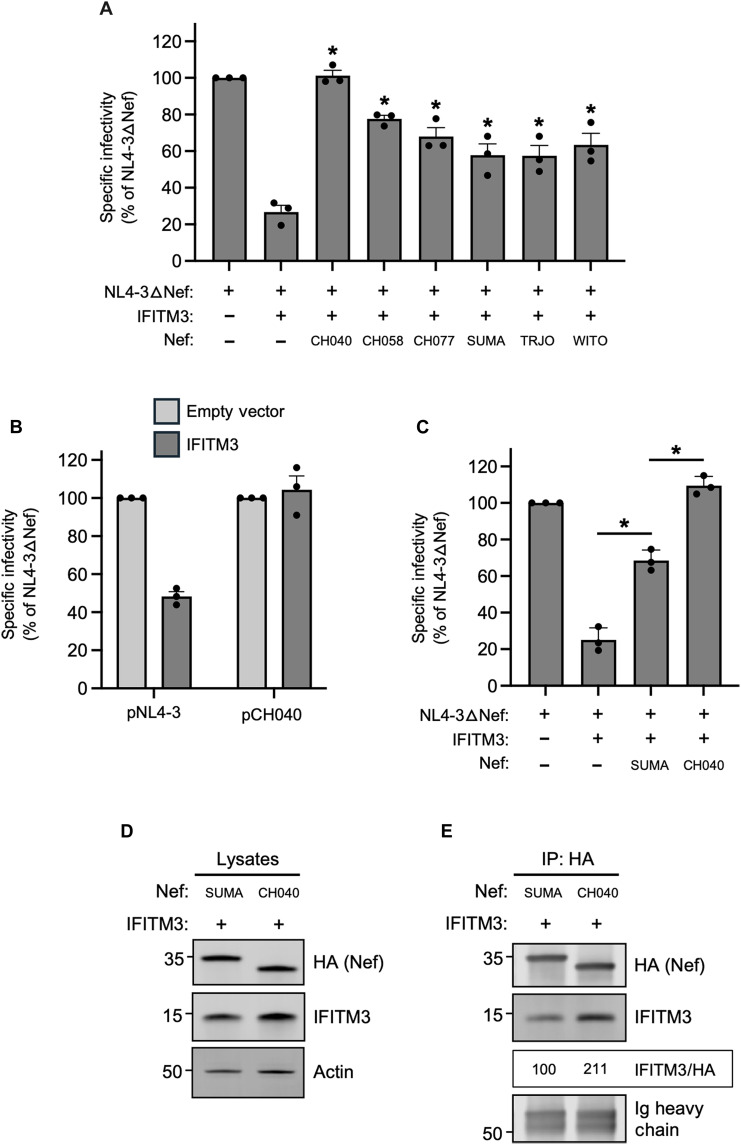
Nef from HIV-1 clade B T/F viruses counteract restriction by IFITM3. (**A**) HEK293T cells were cotransfected with NL4-3△Nef (2.0 μg), pCMV-IFITM3/empty vector (0.5 μg), and pCl-Nef (0.25 μg). Produced virus was harvested 24 hours posttransfection and quantified by p24 ELISA. A 25 ng of p24 equivalents of virus was added to TZM-bl, and infection was scored by anti-Gag immunostaining. Infectivity shown as mean and SD (normalized to NL4-3△Nef alone, set to 100%). Differences between NL4-3△Nef + IFITM3 and the indicated conditions statistically significant by one-way ANOVA (*) (*P* < 0.05). (**B**) HEK293T cells were cotransfected with full-length pNL4-3 or full-length pCH040 (2.0 μg) and pCMV-IFITM3/empty vector (0.5 μg). Produced virus was harvested 24 hours posttransfection and quantified by p24 ELISA. A 25 ng of p24 equivalents of virus was added to TZM-bl, and infection was scored by anti-Gag. Infectivity shown as mean and SD (normalized to empty vector, set to 100%). (**C**) HEK293T cells were cotransfected with NL4-3△Nef (2.0 μg), pCMV-IFITM3 or empty vector (0.5 μg), and pBJ-Nef-HA (0.25 μg). Produced virus was harvested 24 hours posttransfection and quantified by p24 ELISA. A 25 ng of p24 equivalents of virus was added to TZM-bl, and infection was scored by anti-Gag. Infectivity shown as mean and SD (normalized to NL4-3△Nef alone, set to 100%). Differences between NL4-3△Nef + IFITM3 and the indicated conditions statistically significant by one-way ANOVA (*) (*P* < 0.05). (**D**) HEK293T cells were cotransfected with pCMV-IFITM3 (0.50 μg) and pBJ-Nef-HA (0.25 μg), and whole-cell lysates were subjected to SDS-PAGE and immunoblotting. (**E**) From whole-cell lysates in (D), Nef proteins were immunoprecipitated with anti-HA and subjected to SDS-PAGE and immunoblotting (Ig heavy chain served as a loading control). Co-immunoprecipitations were performed twice from independent transfections. The mean IFITM3/HA ratio was calculated for the indicated lanes (normalized to SUMA Nef and IFITM3, set to 100%).

Because we found that primary isolates of Nef obtained from acute clade C, clade D, and clade F HIV-1 infection were capable of counteracting HIV-1 restriction by IFITM3 (Fig. 1A), we next examined Nef proteins from clade B HIV-1, which is the predominant form of HIV-1 in North America. In recent years, much attention has been paid to HIV-1 strains called transmitted/founder (T/F), which represent the phylogenetic ancestors of HIV-1 sequences obtained by single-genome amplification from acutely infected individuals (*47*–*49*). Clade B T/F viruses were previously shown to exhibit elevated infectivity and resistance to interferons relative to viruses obtained during the chronic phase of infection from the same individuals (*49*). We produced six constructs encoding Nef from the T/F viruses CH040, CH058, CH077, SUMA, TRJO, and WITO and tested them for the ability to rescue IFITM3-restricted HIV-1 infectivity. We found that all six T/F Nef proteins counteracted IFITM3, albeit to different extents. The expression of all Nef constructs was detected in transfected cells, and the heightened activity of CH040 Nef relative to the others was not due to increased Nef protein level (fig. S4). Nef proteins from CH040, CH058, and CH077 rescued HIV-1 infectivity to the greatest extent, with CH040 completely restoring HIV-1 infectivity (Fig. 8A). In comparison, the other T/F Nef proteins partially restored infectivity. We also tested full-length CH040 virus for sensitivity to restriction by IFITM3, and we found that it was resistant compared to full length NL4-3 (Fig. 8B). Using additional constructs in which Nef proteins were appended with an HA tag (for the purpose of enabling immunoprecipitation), we confirmed that CH040 Nef completely counteracts IFITM3, while SUMA Nef does so to an intermediate extent (Fig. 8C). To determine whether these two T/F Nef proteins differ with respect to the IFITM3 interaction, we performed immunoprecipitation of Nef and assessed IFITM3 protein pull-down. In accordance with its improved counteraction of IFITM3, CH040 Nef pulled down more IFITM3 protein than SUMA Nef (Fig. 8, D and E). Overall, in addition to the single representatives of HIV-1 clade D, clade F, clade C, and the clade C consensus, at least some clade B T/F viruses encode Nef proteins capable of interacting with and antagonizing the antiviral activity of IFITM3.

## DISCUSSION

Here, we report that restriction of HIV-1 infectivity following ectopic IFITM3 expression in virus-producing cells (epithelial cells or T lymphocytes) is counteracted by Nef proteins obtained from primary isolates of acute HIV-1 infection. In particular, we find that Nef proteins obtained from individuals acutely infected with HIV-1 clade C, the predominant form of circulating HIV-1 worldwide, are especially potent counteractors of IFITM3. Furthermore, Nef proteins that counteracted ectopic IFITM3 also counteracted the antiviral state elicited by type-I interferon treatment. Using clade C Nef to uncover the mechanistic basis for IFITM3 counteraction, we found that Nef interacts with IFITM3, downmodulates IFITM3 from the cell surface, and partially excludes IFITM3 from HIV-1 virions. Furthermore, Nef inhibits IFITM3 oligomerization and reduces the impact of IFITM3 on cellular membrane fluidity, indicating that, in addition to altering the subcellular localization of IFITM3, Nef also functionally inactivates IFITM3. Our findings may suggest that counteraction of IFITM3 by Nef is important during early stages of HIV-1 infection in vivo. This notion is supported by our observation that Nef proteins from clade B T/F viruses also exhibit the capacity to antagonize IFITM3. It will be important to assess whether counteraction of IFITM3 is also a feature of Nef proteins isolated during chronic stages of HIV-1 infection. Nonetheless, it is possible that this previously uncharacterized activity of Nef is particularly important in the context of the interferon-induced antiviral state, for example, during the initial infection of and virus spread within mucosal tissue following virus transmission.

While performing the experiments detailed in this manuscript, it was reported that HIV-1 Nef reduces the levels of IFITM proteins found in extracellular vesicles released from T cells (*31*). Mechanistically, Nef downmodulated constitutively expressed human IFITM1, IFITM2, and IFITM3 from the surface of T cells and reduced IFITM1 to IFITM3 levels in detergent-resistant lipid rafts. Our findings presented here, in the context of HIV-1 infection, corroborate those data in that primary isolates of Nef counteracted IFITM3 antiviral function in a manner associated with its downmodulation from the cell surface. While the majority of our work was performed using HEK293T epithelial cells as virus-producing cells, we also found that Nef countered ectopic IFITM3 and boosted HIV-1 infectivity when using a doxycycline-inducible SupT1 lymphocyte cell line. It will be of interest to address whether Nef influences the localization of IFITM3 to lipid rafts in virus-producing cells. Early studies on human IFITM proteins implicated them as part of signaling complexes associated with lipid rafts (*50*, *51*). Palmitoylation is a posttranslational modification that promotes the localization of transmembrane proteins to lipid rafts (*52*). Human IFITM3 is palmitoylated at conserved cysteine residues, and this lipidation promotes membrane anchoring and promotes antiviral functions (*27*, *53*, *54*) including the restriction of HIV-1 (*27*). IFITM3 also directly interacts with membrane cholesterol (*55*–*57*), and its inhibition of membrane fluidity may depend on cholesterol (*58*). Nef was reported to be a cholesterol-binding, lipid raft–localizing protein, and these traits are associated with its ability to promote HIV-1 infectivity (*59*). Since egress from cholesterol-enriched lipid rafts in virus-producing cells is important for HIV-1 infectivity (*60*–*64*), it is possible that raft-associated IFITM3 reduces the level of cholesterol available to budding virions, while Nef restores cholesterol levels there, possibly by excluding IFITM3 from rafts.

HIV-1 was previously reported to boost HIV-1 infectivity by counteracting the antiviral activities of SERINC3/5, which are not regulated by interferons (*9*, *10*). In that sense, our demonstration that primary isolates of Nef can counteract IFITM3 may represent a rare instance of HIV-1 Nef targeting an interferon-stimulated gene product. HIV-1 Nef has been reported to regulate the production of interferon by interfering with signal transducers and activators of transcription 1 phosphorylation (*14*), but less is known about the direct antagonism of interferon-stimulated effector proteins by Nef. Another HIV-1 accessory protein, Vpu, antagonizes the interferon-inducible protein BST2/tetherin (*65*, *66*), but in certain nonhuman lentiviruses that lack a Vpu open reading frame, such as SIV, antagonism of tetherin is performed by Nef (*67*, *68*). Furthermore, it was shown that certain isolates of HIV-1 group M encode Nef proteins with the functional capacity to counteract human tetherin (*69*). Therefore, our work here indicates that IFITM3 can be added to the short list of interferon-stimulated gene products targeted by HIV-1 Nef.

It is worth noting that the mechanisms used by Nef to counteract SERINC3/5 and IFITM3 are similar yet distinct. This is demonstrated by the sensitivity of SERINC3/5 to different Nef proteins as compared to the sensitivity of IFITM3 to those same Nef proteins. It was previously shown that Nef from the clade H isolate 90CF056 poorly counteracts SERINC3/5 (*10*, *70*), while Nef from clade C isolate 97ZA is a more potent antagonist (*10*). This matches the effects of those same Nef proteins on IFITM3-mediated restriction (Fig. 1A). However, 97ZA Nef completely restores HIV-1 infectivity in the presence of IFITM3 but only partially restores infectivity in the presence of SERINC5 (Fig. 2E). Furthermore, SF2 Nef counteracts SERINC5 to a similar extent as 97ZA Nef (Fig. 2E) (*10*), whereas these Nef proteins differentially counteract IFITM3 (Fig. 2A). These results indicate that the sequence determinants used by Nef to counteract SERINC3/5 versus those used to counteract IFITM3 are nonidentical. We found that the C terminus of Nef dictates the degree to which both IFITM3 interaction and IFITM3 counteraction occur (Fig. 6, A to C). More specifically, we identified the C-terminal residues S152 and E153 as being highly consequential for IFITM3 binding and its counteraction (Fig. 7, A to C). Future work will highlight the residues in IFITM3 that are targeted by the C terminus of Nef to gain a greater understanding of the IFITM3-Nef binding interface, which may involve other cellular proteins such as AP-2. In that regard, recombinant IFITM3, Nef, and AP-2 proteins could be used to measure the direct binding potential between these proteins, and the structural characterization may help illuminate the protein-protein and protein-protein-protein interfaces involved. Furthermore, it will be interesting and important to establish the species-specific nature (or lack thereof) of the IFITM3-Nef relationship. For example, determining whether nonhuman IFITM3 proteins are sensitive to HIV-1 Nef and whether human IFITM3 is sensitive to Nef from other primate lentiviruses may reveal additional details about the IFITM3-Nef functional interface and will determine whether coevolution occurs at this host-virus interaction as part of an ongoing genetic conflict.

Our results suggest that Nef may boost HIV-1 infectivity by targeting multiple cellular factors, including IFITM3 and SERINC3/5, that restrict HIV-1 infectivity. It remains to be determined whether IFITM3, or a related IFITM protein, represents the unknown target of Nef that limits infectivity in MOLT-3 and CEM T cell lines in a SERINC-independent manner (*23*–*25*). However, because these reports almost exclusively used laboratory-adapted NL4-3 Nef protein—a poor counteractor of IFITM3 in our study—it seems unlikely. Therefore, it is plausible that there exist additional Nef-sensitive factors limiting HIV-1 infectivity, which remain to be identified.

## MATERIALS AND METHODS

### Cells and cell culture reagents

HEK293T (American Type Culture Collection, CRL-3216), TZM-bl (NIH HIV Reagents Program/BEI; HRP-8129) and HT1080-mCAT1 [described previously (*71*)] were cultured in Dulbecco’s modified Eagle’s medium (DMEM; Gibco) supplemented with 10% heat-inactivated fetal bovine serum (FBS; Hyclone) and 1% penicillin-streptomycin (Gibco) at 37°C with 5% CO_2_. HEK293T stably expressing empty vector and IFITM3 were previously described (*32*). Tet ON SupT1-IFITM3 cells were previously described (*26*, *72*) and were cultured in RPMI (Gibco) supplemented with 10% heat-inactivated FBS (Hyclone) and 1% penicillin-streptomycin (Gibco) at 37°C with 5% CO_2_. Doxycycline hyclate was obtained from Sigma-Aldrich (D5207). Human interferon-β1a was obtained from PBL Assay Science (11415-1).

### Plasmids and molecular biology

pCMV-hIFITM3, pCMV-hIFITM2, and empty vector were obtained from OriGene. pBJ-97ZA Nef-HA, pBJ-90CF Nef-HA, pBJ-93BR Nef-HA, pBJ-94UG Nef-HA, pBJ-SF2 Nef-HA, pBJ-LAI Nef-HA, and pBJ-SERINC5 were obtained from H. Gottlinger (University of Massachusetts). pCl-CH040 Nef, pCl-THRO Nef, pCl-WITO Nef, pCl-CH077 Nef, pCl-CH058 Nef, pCl-TRJO Nef, and pCl-SUMA Nef were obtained from Mary Lewinski and John Guatelli (University of California San Diego). pBJ-NL4-3 Nef-HA, pBJ-CH040 Nef-HA, pBJ-SUMA Nef-HA, chimeric pBJ-NL4-3/97ZA Nef-HA and pBJ-97ZA/NL4-3 Nef-HA, and mutants of pBJ-97ZA Nef-HA were produced in this study by PCR amplification and Bam H1/Eco R1 restriction sites. A consensus Nef derived from 105 acute HIV-1 clade C infections (*35*) was generated online (www.hiv.lanl.gov/content/sequence/CONSENSUS/SimpCon.html) and cloned into pBJ to produce the pBJ-Con C Nef-HA construct. The following full-length virus constructs, and their sources, are as follows: pNL4-3 (Olivier Schwartz, Pasteur Institute); NL4-3△Nef (Olivier Schwartz, Pasteur Institute); pCH040 (Nicoletta Casartelli and Olivier Schwartz, Pasteur Institute); pNL4-3 encoding 90CF, 93BR, 94UG, and 97ZA Nef (Heinrich Gottlinger, University of Massachusetts); pNL4-3 encoding HIV-2 BEN Nef or SIVmac239 Nef (R. Sloan, University of Edinburgh) (*73*). The plasmids encoding glycoGag-deficient Moloney MLV Gag-Pol, pCMV-glycoGag-Myc, pBabeLuc, and pCMV-Xenogp85 (xenotropic Env) were previously described (*30*). EEA1-GFP was obtained from Addgene (42307). A list of Nef protein sequences used in this study is found in file S1.

### Virus production and infections

For virus production in HEK293T, cells were seeded (200,000 cells per well) in a six-well plate and transfected with the indicated viral plasmids using Mirus TransIT-293 transfection reagent (Mirus Bio, 2700). Virus containing supernatants were harvested at 24 hours posttransfection and centrifuged at 2500 rpm for 5 min to remove cellular debris. HIV-1 quantity was measured using the HIV-1 p24 ELISA Kit (XpressBio, XB-1000), while MLV quantity was measured using anti-p30 immunoblotting. To measure specific HIV-1 infectivity, 25 ng of p24 equivalents was added to TZM-bl cells. Cells were fixed/permeabilized with Cytofix/Cytoperm Solution (BD, 554722) at 48 hours postinfection and immunostained with anti-Gag KC57–fluorescein isothiocyanate (FITC) (Beckman Coulter, 6604665). Samples were acquired on a BD LSRFortessa flow cytometer and analyzed using FlowJo software (version 10.8.1). To measure MLV infectivity, virus was added to HT1080-mCAT1 cells, and infection was measured at 48 hours postinfection using luciferase assay (Promega, E1501).

For virus production in Tet-ON SupT1-IFITM3 cells, 10,000 cells were seeded in a 48-well plate and inoculated with 200 ng p24 equivalents of NL4-3△Nef or NL4-3(97ZA Nef). Eighteen hours later, cells were washed with PBS and resuspended in fresh RPMI containing doxycycline (500 ng/ml) or not. At 3 days doxycycline addition, cells were spun down, virus-containing supernatants were harvested, and virus was quantified by p24 ELISA. A 75 ng of p24 equivalents was added to TZM-bl cells, and cells were fixed at 48 hours postinfection and immunostained with KC57-FITC to enumerate infection by flow cytometry.

For virus entry/fusion measurements, HIV-1 produced from HEK293T cells was labeled with SP-DiOC18 (Thermo Fisher Scientific, D7778) at a final concentration of 0.2 μM for 1 hour at room temperature. Labeled viruses were filtered through 0.22-μm filters to remove virus aggregates, and virus quantity was measured using HIV-1 p24 ELISA Kit (XpressBio, XB-1000). A 40 ng of p24 equivalents of labeled virus was added to TZM-bl cells seeded (80,000 cells per well) in an eight-well Mu-slide dish (Ibidi, 80826), and cells were incubated for 1 hour on ice. Cells were washed three times with serum-free DMEM; fresh, serum-containing DMEM was added to cells; and cells were incubated for 1 hour at 37°C. Cells were then fixed with 4% formaldehyde for 15 min at 4°C. Nuclei were labeled with Hoechst 33342 (Thermo Fisher Scientific, 62249) for 10 min at room temperature. The T-20 fusion inhibitor was obtained from the NIH HIV Reagent Program (9845). SP-DiOC18 fluorescence was measured imaged on a Leica Stellaris confocal fluorescence microscope. Images were analyzed and SP-DiOC18 fluorescence intensities were measured by calculating integrated densities (a measurement of fluorescence intensity that sums pixel values within a selected region of interest) within regions of interest encompassing 8 to 15 cells per condition in Fiji (ImageJ).

### Flow cytometry

For quantification of HIV-1 infection, TZM-bl cells were fixed with Cytofix/Cytoperm solution (BD, 554722) and stained with Anti-HIV-1 Core Antigen KC57-FITC (Beckman Coulter, 6604665) for 30 min at room temperature. For cell surface staining of IFITM3, transfected HEK293T cells were stained with anti-IFITM3 (Proteintech, 66081-1) on ice for 30 min and then fixed with 2% formaldehyde solution (Invitrogen, FB002) for 10 min at room temperature. Cells were then washed with PBS and stained with secondary antibody goat anti-mouse IgG (H + L) Alexa Fluor 488 (Invitrogen, A11001).

For staining of total IFITM3 in Tet ON SupT1-IFITM3 cells, cells were fixed with Cytofix/Cytoperm solution (BD, 554722) and stained with anti-IFITM3 (Abcam, ab109429). Cells were washed with Cyto Perm/Wash buffer (BD, 554723) and stained with goat anti-rabbit IgG (H + L) Alexa Fluor 647. Cells were analyzed on a BD LSRFortessa flow cytometer and analyzed using FlowJo software (versions 10.8.1).

### Western blot analysis

Whole-cell lysis was performed using a buffer consisting of 20 mM Hepes, 150 mM NaCl, 1 mM EDTA, and 1% Triton X-100 (Sigma-Aldrich, X100) containing Halt protease inhibitor cocktail, EDTA free (Thermo Fisher Scientific, 78425). Lysis was performed on ice for 30 min before centrifugation at 12,000 rpm for 10 min at 4°C, and supernatants were harvested. The protein quantity was measured using the Protein Assay (Bio-Rad, 5000001). Lysates were mixed with NuPAGE Reducing Agent (Invitrogen, NP0009) and Protein Loading Buffer (Li-COR, 928-40004) and loaded into Criterion XT 12% polyacrylamide bis-tris gels (Bio-Rad, 3450117). SDS–polyacrylamide gel electrophoresis (SDS-PAGE) was performed with NuPAGE MES SDS Running Buffer (Invitrogen, NP0002). Proteins were transferred to Immobilion polyvinylidene difluoride membrane and a pore size of 0.45 μm (Millipore, IPFL00010). Membranes were blocked with Intercept PBS Blocking Buffer (Li-COR, 927-70001) for 30 min at room temperature. The following primary antibodies were used: anti-IFITM3 (Abcam, ab109429), anti-IFITM2 (Proteintech, 66137-1-Ig), anti-HA (Abcam, ab9110), anti-Actin (Santa Cruz Biotechnology, sc-47778), anti-Nef (NIH HIV Reagent Program/BEI, ARP-2949), anti-Gag (183-H12-5C) (NIH HIV Reagent Program/BEI, ARP-3537), anti-FLAG (M2) (Sigma-Aldrich, F1804), anti-Myc (Sigma-Aldrich, C3956), anti-syntenin (Abcam, ab133267), and anti-EEA1 (BD Biosciences, 610456). The following secondary antibodies were used: goat anti-mouse IRDye 800CW (Li-COR, 926-32210), goat anti-mouse IRDye 680RD (Li-COR, 926-68070), goat anti-rabbit 800CW (Li-COR, 926-32211), and goat anti-rabbit 680RD (Li-COR, 926-68071). Images were obtained with the Li-COR Odyssey CLx, and analysis was performed with ImageStudio Lite software (Li-COR). PageRuler Prestained Protein Ladder, 10 to 180 kDa, was used as protein standard (Thermo Fisher Scientific, 26616).

### Co-immunoprecipitation

Anti-HA antibody (Abcam, ab9110) or anti-FLAG antibody (M2) (Sigma-Aldrich, F1804) was added to 100 μg of whole-cell lysates, and the protein-antibody mixture was incubated for 1 hour at 4°C. A 10 μl of Dynabeads Protein G (Invitrogen, 10007D) was added to the protein-antibody mixture for 1 hour at 4°C, and reaction mixtures were centrifuged at 1000*g* for 3 min at 4°C. The supernatant was removed, and the pelleted beads were washed three times with cell lysis buffer. The bead fraction was resuspended with NuPAGE Reducing Agent (Invitrogen, NP0009) and Protein Loading Buffer (Li-COR, 928-40004), boiled for 5 min at 95°C, and loaded into Criterion XT 12% polyacrylamide bis-tris gels (Bio-Rad, 3450117). SDS-PAGE and Western blot analysis were performed as described above.

### Proximity ligation assay

In situ PLA was performed with the Duolink In Situ Red Starter Kit Mouse/Rabbit (Sigma-Aldrich, DUO92101) according to the manufacturer’s protocol. HEK293T cells were transfected and seeded (50,000 cells per well) in eight-well Mu-slide dish (Ibidi, 80826), fixed/permeabilized with Cytofix/Cytoperm solution (BD, B554714) for 5 min, and blocked with Duolink Blocking Solution (1×) for 1 hour at 37°C. Cells were then incubated with primary antibodies [rabbit anti-IFITM3 (Abcam, ab109429) and mouse anti-HA (BioLegend, 901533)] for 1 hour at room temperature. Cells were washed twice with buffer A and subsequently incubated with the probes affinity purified Donkey anti-Rabbit IgG (anti-Rabbit PLUS, Sigma-Aldrich, DUO92002) and affinity purified Donkey anti-Mouse IgG (anti-Mouse PLUS, Sigma-Aldrich, DUO92004) for 1 hour at 37°C. After washing cells twice with buffer A, oligonucleotide ligation was performed for 30 min at 37°C. Cells were washed two additional times with buffer A, followed by incubation with amplification stock solution for 100 min at 37°C. After washing twice with buffer B, Hoechst 33342 (Thermo Fisher Scientific, H3570) was added for 5 min at room temperature to label nuclei. Image acquisition was performed with a Leica Stellaris confocal fluorescence microscope. Images were analyzed and processed using Fiji (ImageJ). Fluorescence intensities were quantified by calculating the mean per-cell corrected total cell fluorescence (CTCF) from regions of interest encompassing 6 to 12 cells per condition. CTCF values are derived by calculating the integrated density (a measurement of fluorescence intensity that sums pixel values within a selected region of interest), subtracting background fluorescence, and correcting for the area of the region of interest.

### Confocal immunofluorescence microscopy

HEK293T were transfected and seeded (25,000 cells per well) in an eight-well Mu-slide dish (Ibidi, 80826), fixed/permeabilized with Cytofix/Cytoperm solution (BD, 554722) for 5 min, and blocked with 3% bovine serum albumin. Cells were then incubated with primary antibodies [rabbit anti-IFITM3 (Abcam, ab109429) and mouse anti-HA (BioLegend, 901533)] for 1 hour at room temperature. After washing twice with Cyto Perm/Wash Solution (BD, 554723), cells were incubated with secondary antibodies [goat anti-mouse IgG (H + L) Secondary Antibody, Alexa Fluor 555 and goat anti-rabbit IgG (H + L) Secondary Antibody, Alexa Fluor 647)]. Nuclei were labeled with Hoechst 33342, and image acquisition was performed using a Leica Stellaris confocal microscope. Images were analyzed and processed using Fiji (ImageJ).

### Measurement of membrane fluidity by FLIM

HEK293T stably expressing IFITM3 or empty vector (previously described (*32*)) were untransfected or transfected with pBJ-97ZA Nef-HA (0.25 μg). MBCD (Sigma-Aldrich, C4555) was added to HEK293T-IFITM3 cells at a final concentration of 5 mM for 1 hour at 37°C. Flipper-TR probe (Cytoskeleton, CY-SC020) was added to cells at a final concentration of 1 μM, in serum-free media, for 10 min at 37°C. Medium was replaced with FluoroBrite DMEM supplemented with 1% penicillin-streptomycin and 10% FBS, and imaging was performed on a Zeiss AG 880 laser confocal fluorescence microscope equipped with a live cell imaging environmental chamber and a Coherent Chameleon Vision II pulsed near-infrared laser used for two-photon excitation, in conjunction with SPCM 64 fluorescence lifetime imaging (FLIM) acquisition software. Lifetimes were detected with an HPM-100-40 detector (Becker & Hickl), and collection time was set to 60 s using SPC-QC-104 timing electronics (Becker & Hickl). The analysis was performed with SPCImage 8.8 software (Becker & Hickl). Raw images were uploaded, and parameters were set as follows: spatial binning of 2, threshold of 6, and single exponential decay analysis. A region of interest tracing the periphery of each cell was established, and the lifetime of all pixels within the region of interest was averaged. Analyzed images were scaled, from red to blue, on a range of 1800 to 3800 ps.

### Statistical analysis

Tests for statistical significance were performed in GraphPad Prism.
