# Supplementary material for: Restriction of HIV-1 infectivity by interferon and IFITM3 is counteracted by Nef
Source: Sci Adv. 2025 Oct 1;11(40):eadz7083. doi: 10.1126/sciadv.adz7083 (PMC12487890; doi:10.1126/sciadv.adz7083)
Supplement: Supplementary file 1 — Figs. S1 to S4 File S1 [file sciadv.adz7083_sm.pdf]

Science Advances
AAAS

Supplementary Materials for

Restriction of HIV-1 infectivity by interferon and IFITM3 is counteracted by Nef

Mahesh Agarwal *et al.*

Corresponding author: Alex A. Compton, alex.compton@nih.gov

Sci. Adv. **11**, eadz7083 (2025)
DOI: 10.1126/sciadv.adz7083

This PDF file includes:

Figs. S1 to S4
File S1

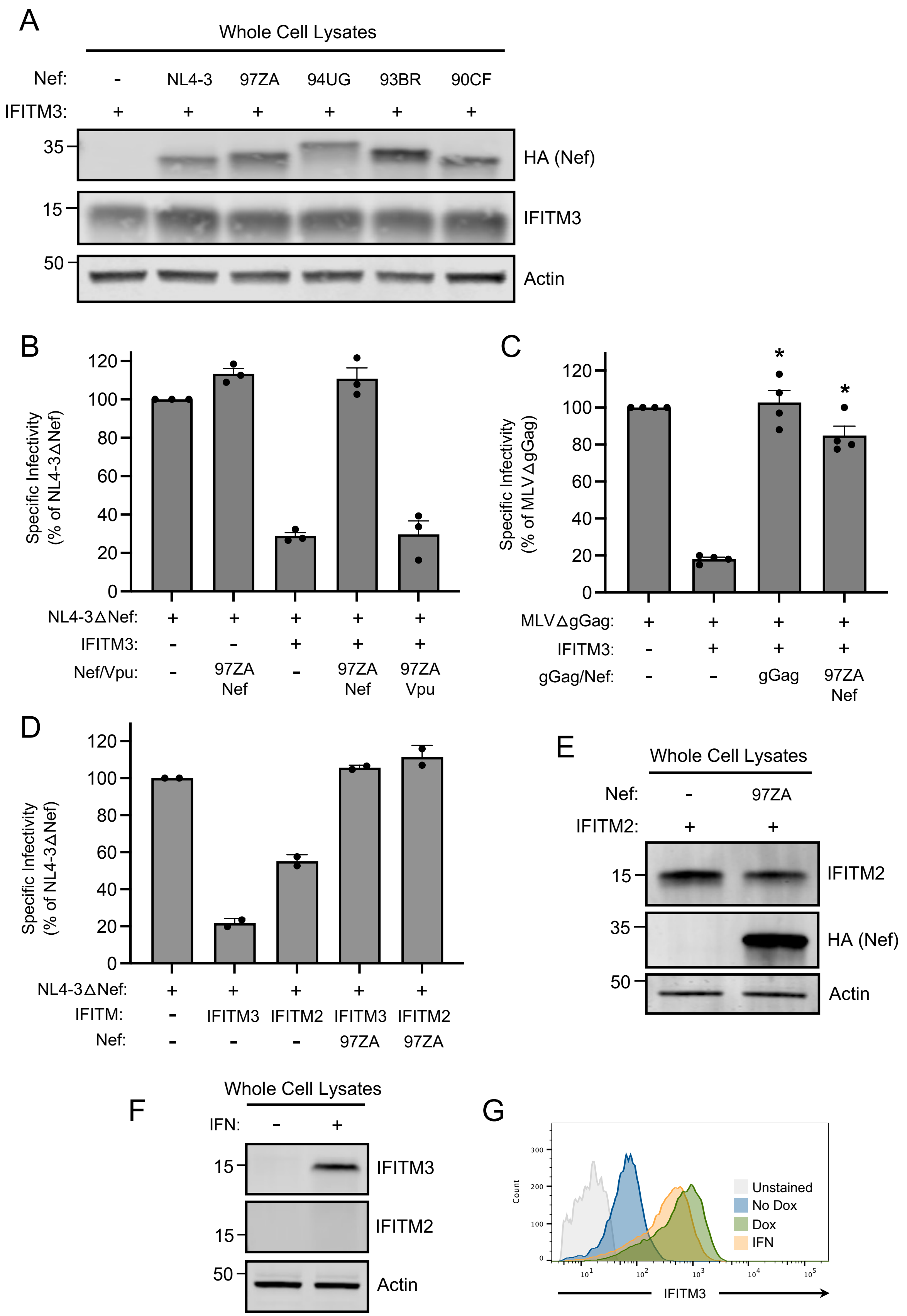

A
Whole Cell Lysates
Nef:
-
NL4-3
97ZA
94UG
93BR
90CF
IFITM3:
+
35
15
50
HA (Nef)
IFITM3
Actin
B
Specific Infectivity
(% of NL4-3ΔNef)
0
20
40
60
80
100
120
NL4-3ΔNef:
IFITM3:
Nef/Vpu:
97ZA Nef
97ZA Nef
97ZA Vpu
C
Specific Infectivity
(% of MLVΔgGag)
*
*
MLVΔgGag:
IFITM3:
gGag/Nef:
gGag
97ZA Nef
D
Specific Infectivity
(% of NL4-3ΔNef)
NL4-3ΔNef:
IFITM:
IFITM3
IFITM2
IFITM3
IFITM2
Nef:
97ZA
97ZA
E
Whole Cell Lysates
Nef:
-
97ZA
IFITM2:
+
15
35
50
IFITM2
HA (Nef)
Actin
F
Whole Cell Lysates
IFN:
-
+
15
15
50
IFITM3
IFITM2
Actin
G
Count
300
200
100
0
Unstained
No Dox
Dox
IFN
IFITM3

Supplemental Figure 1: Nef, but not Vpu, counteracts IFITM3 and IFITM2 in the context of HIV and MLV infection.

(A) HEK293T were co-transfected with pCMV-IFITM3 (0.50 μg) and pBJ-Nef-HA encoding the indicate Nef (0.25 μg), and whole cell lysates were subjected to SDS-PAGE and immunoblotting with anti-HA, anti-IFITM3, and anti-Actin (B) HEK293T were co-transfected with NL4-3ΔNef (2.0 μg), pCMV-IFITM3 or Empty Vector (0.5 μg), and pBJ-97ZA Nef-HA or pBJ-97ZA Vpu-HA (0.25 μg). Produced virus was harvested 24 hours post-transfection and quantified by p24 ELISA. 25 ng p24 equivalents of virus were added to TZM-bl, and infection was scored by anti-Gag immunostaining. Infectivity is shown as mean and standard deviation (normalized relative to NL4-3ΔNef alone, set to 100%). Filled circles represent biological replicates (independent transfections). (C) HEK293T were co-transfected with MLVΔglycoGag (2.5 μg), pBabeLuc (0.6 μg), pCMV-Xenogp85 (xenotropic Env) (0.5 μg), and pCMV-IFITM3 or Empty Vector (0.5 μg), and where indicated, pCMV-glycoGag-Myc (0.25 μg) or pBJ-97ZA Nef-HA (0.25 μg). Produced virus was harvested 48 hours post-transfection and quantified by viral Gag immunoblotting with anti-p30 of pelleted viruses. Equal volumes of p24 equivalent of virus were added to HT1080-mCAT1cells, and infection was scored by luciferase assay at 48 hours post-inoculation. Luciferase values were divided by Gag immunoblot intensity to derive a specific infectivity measurement. Virus infectivity of each condition is shown as mean and standard deviation (normalized relative to MLVΔglycoGag alone, set to 100%). Differences statistically different by one way ANOVA from the indicated condition and MLVΔglycoGag + IFITM3 are indicated by (*) ($p < 0.05$). (D) HEK293T were co-transfected with NL4-3ΔNef (2.0 μg), pCMV-IFITM3 or pCMV-IFITM2 or Empty Vector (0.5 μg), and pBJ-97ZA Nef-HA (0.25 μg). Produced virus was harvested 24 hours post-transfection and quantified by p24 ELISA. 25 ng p24 equivalents of virus was added to TZM-bl, and infection was scored by anti-Gag immunostaining. Infectivity is shown as mean and standard deviation (normalized relative to NL4-3ΔNef alone, set to 100%). (E) HEK293T were co-transfected with pCMV-IFITM2 (0.50 μg) and pBJ-97ZA Nef-HA (0.25 μg), and whole cell lysates were subjected to SDS-PAGE and immunoblotting with anti-HA, anti-IFITM2, and anti-Actin. (F) HEK293T were untreated or treated with ~30 units type-I IFN (IFN Beta 1a) for 18 hours and whole cell lysates were subjected to SDS-PAGE and immunoblotting with anti-IFITM3, anti-IFITM2, and anti-Actin. (G) SupT1 Tet-On IFITM3 (SupT1-IFITM3) cells were untreated, treated with doxycycline (500 ng/mL), or treated with type-I IFN (IFN Beta 1a) (~30 units) for 18 hours. Cells were fixed, immunostained with anti-IFITM3 antibody and analyzed with flow cytometry. Unstained cells served as control for background fluorescence.

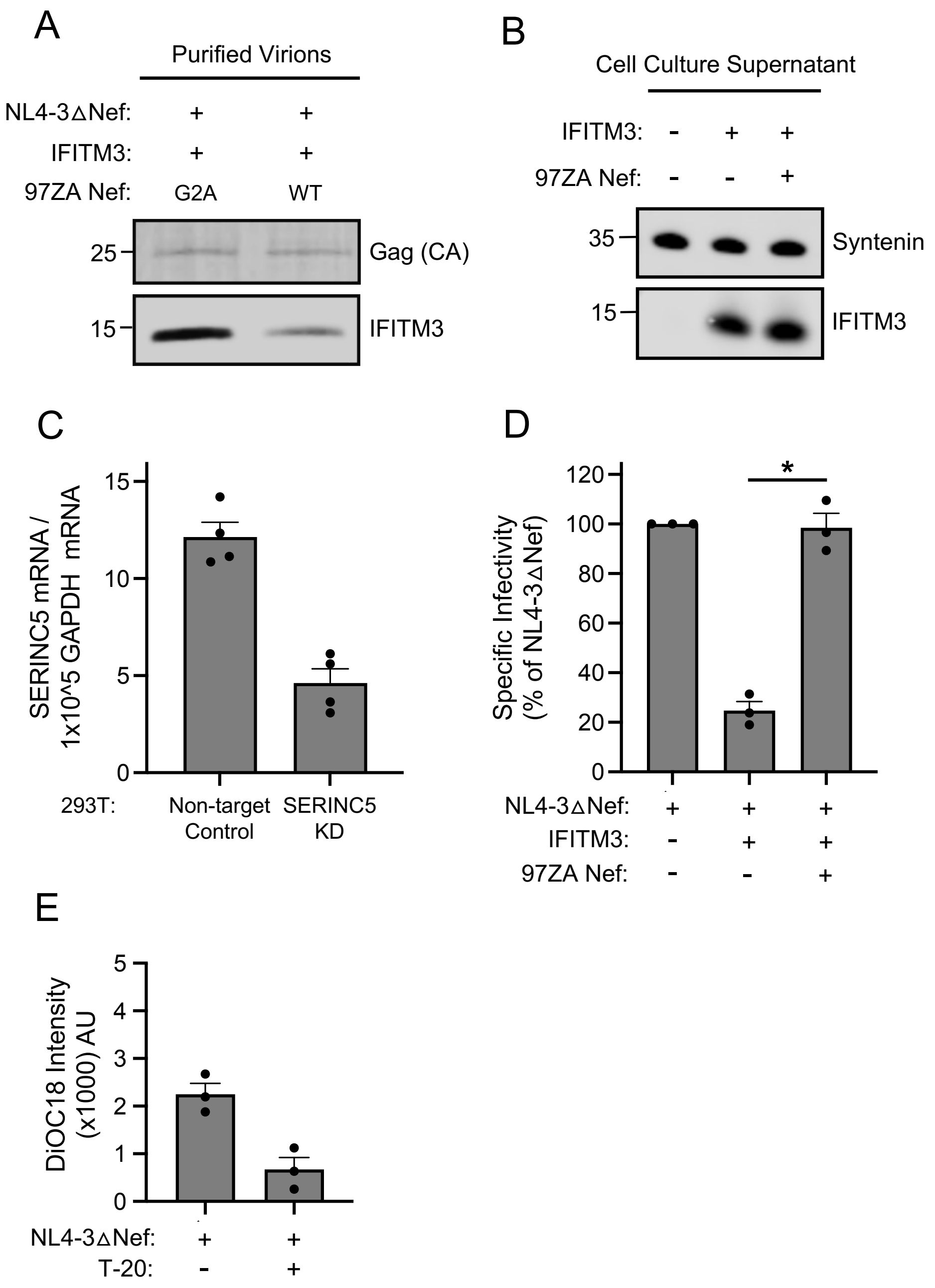

A
Purified Virions
NL4-3△Nef: + +
IFITM3: + +
97ZA Nef: G2A WT
25
Gag (CA)
15
IFITM3
B
Cell Culture Supernatant
IFITM3: - + +
97ZA Nef: - - +
35
Syntenin
15
IFITM3
C
SERINC5 mRNA / 1x10^5 GAPDH mRNA
15
10
5
0
293T:
Non-target Control
SERINC5 KD
D
Specific Infectivity (% of NL4-3△Nef)
*
120
100
80
60
40
20
0
NL4-3△Nef: + + +
IFITM3: - + +
97ZA Nef: - - +
E
DiOC18 Intensity (x1000) AU
5
4
3
2
1
0
NL4-3△Nef: + +
T-20: - +

Supplemental Figure 2: Nef counteracts IFITM3 in a SERINC5-independent manner by reducing the incorporation of IFITM3 into HIV-1 virions but not extracellular vesicles.
(A) HEK293T were co-transfected with NL4-3ΔNef (2.0 μg), pCMV-IFITM3 (0.5 μg), and pBJ-Nef-HA encoding the indicated Nef (0.25 μg). Produced virus was harvested 24 hours post-transfection, passed through a 0.45 micron filter, purified by ultracentrifugation over a 20% sucrose cushion at 25,000 rpm for 1 hour, and quantified by p24 ELISA. 10 ng p24 equivalents were subjected to SDS-PAGE and immunoblotting with anti-Gag and anti-IFITM3. (B) HEK293T were co-transfected with pCMV-IFITM3 or Empty Vector (0.5 μg) and pBJ-97ZA Nef-HA (0.25 μg) in the indicated condition. Cell culture media were harvested 24 hours post-transfection and ultracentrifuged at 25,000 rpm for 1 hour. Equivalent volumes of concentrated media were subjected to SDS-PAGE and immunoblotting with anti-IFITM3 and anti-Syntenin. Syntenin is a marker for extracellular vesicles. (C) Stable SERINC5 knockdown was performed in HEK293T cells as previously described (74) and knockdown was assessed by quantitative RT-PCR. SERINC5 mRNA levels per 1x10^5 copies of GAPDH mRNA were plotted. (D) SERINC5 knockdown HEK293T cells were co-transfected with NL4-3ΔNef (2.0 μg), pCMV-IFITM3 or Empty Vector (0.5 μg), and pBJ-97ZA Nef-HA (0.25 μg). Produced virus was harvested 24 hours post-transfection and quantified by p24 ELISA. 25 ng p24 equivalents of virus were added to TZM-bl, and infection was scored by anti-Gag immunostaining. Infectivity is shown as mean and standard deviation (normalized relative to NL4-3ΔNef alone, set to 100%). Differences between the indicated conditions statistically significant by student's T test are indicated by (*) ($p < 0.05$). Filled circles represent biological replicates (independent transfections). (E) HEK293T were co-transfected with NL4-3ΔNef (2.0 μg). Produced virus was harvested 24 hours post-transfection and one mL was labeled with SP-DiOC18 at a final concentration of 0.2 μM for 60 minutes at room temperature. Labeled virus quantity was measured using p24 ELISA. 40 ng p24 equivalents of labeled virus were added to TZM-bl on ice for 1 hour, in the absence or presence of 20 μg/mL T-20, and then cells were incubated at 37°C for 1 hour and subsequently fixed. Nuclei were stained with Hoechst and confocal fluorescence microscopy was performed. DiOC18 fluorescence intensity in each condition is shown as means and standard deviation. Filled circles represent fields of view containing 6-12 cells each.

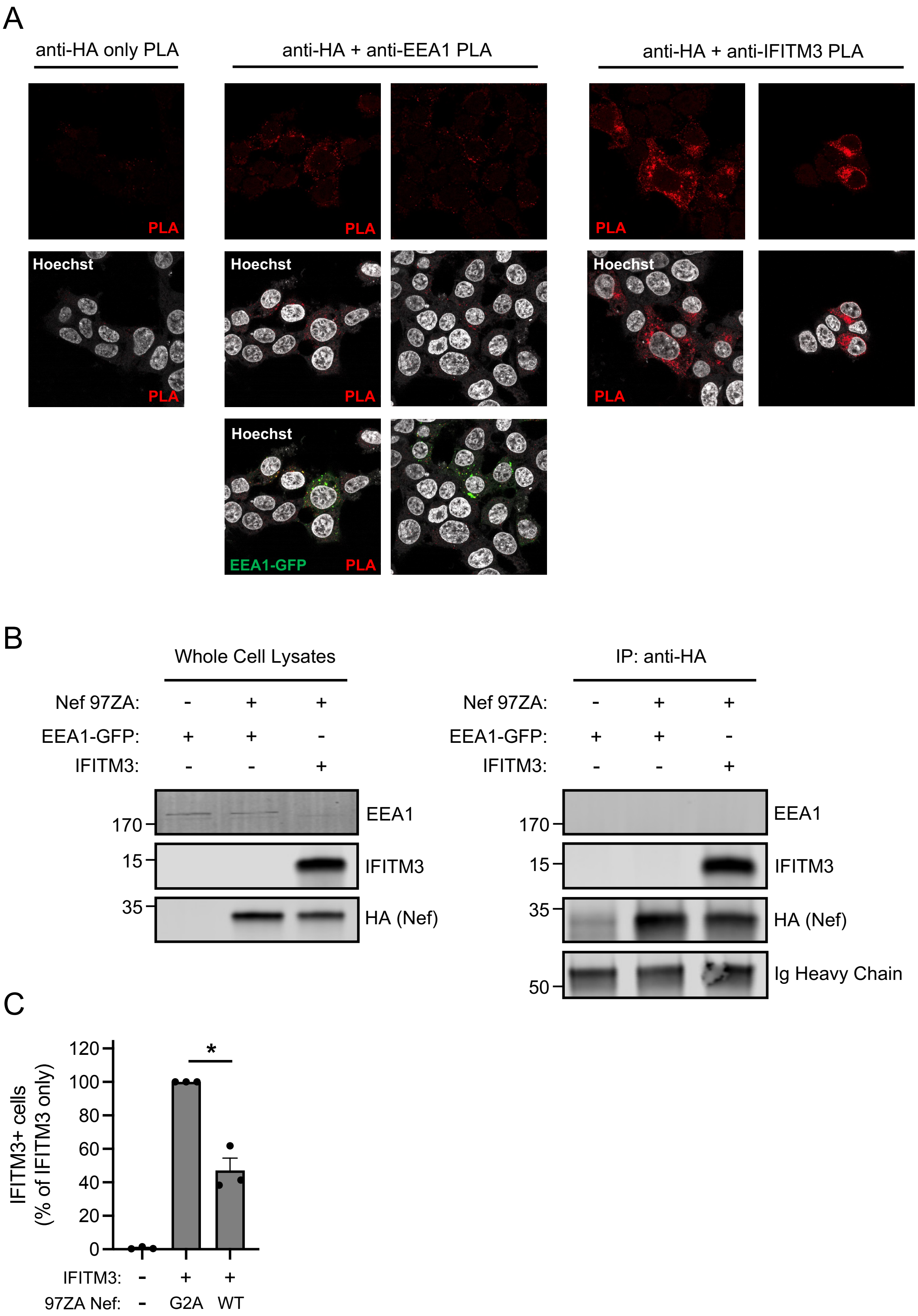
A
anti-HA only PLA
anti-HA + anti-EEA1 PLA
anti-HA + anti-IFITM3 PLA
PLA
Hoechst
EEA1-GFP
B
Whole Cell Lysates
IP: anti-HA
Nef 97ZA: - + +
EEA1-GFP: + + -
IFITM3: - - +
EEA1
IFITM3
HA (Nef)
Ig Heavy Chain
170
15
35
50
C
IFITM3+ cells (% of IFITM3 only)
0
20
40
60
80
100
120
*
IFITM3: - + +
97ZA Nef: - G2A WT

Supplemental Figure 3: Nef selectively interacts with IFITM3 in membranes.
(A) HEK293T were co-transfected with pBJ-97ZA Nef-HA (0.25 µg) and either pCMV-IFITM3 or EEA1-GFP (0.50 µg). Cells were fixed at 24 hours post-transfection and proximity ligation assay was performed using anti-IFITM3 and anti-HA or anti-EEA1 and anti-HA followed by confocal microscopy. Nuclei were stained with Hoechst. A negative control consisting of anti-HA alone was performed to establish background fluorescence. (B) HEK293T were co-transfected as in (A) and Nef proteins were immunoprecipitated with anti-HA followed by SDS-PAGE and immunoblotting with anti-HA, anti-EEA1, and anti-IFITM3. Ig heavy chain served as loading control. (C) HEK293T were transfected with pCMV-IFITM3 (0.50 µg) alone or both pCMV-IFITM3 and pBJ-97ZA Nef-HA (WT or G2A) (0.25 µg) and living, intact cells were stained with anti-IFITM3. Subsequently, cells were fixed, and IFITM3-positive cells were quantified by flow cytometry. Summary data of three biological replicates (independent transfections) is shown as means and standard deviation (normalized to cells transfected with IFITM3 alone, set to 100%). Differences between the indicated conditions statistically significant by student's T test are indicated by (*) ($p < 0.05$).

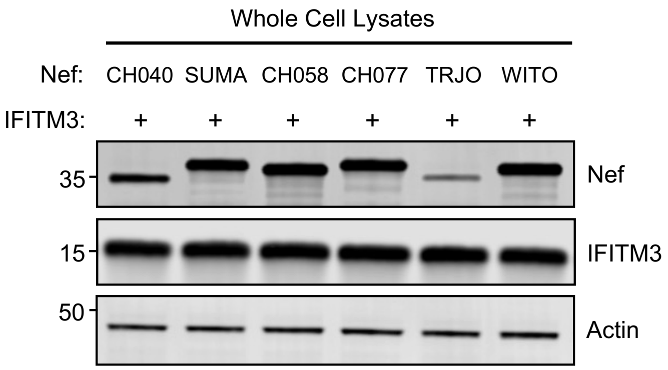

Supplemental Figure 4: Nef proteins from HIV-1 transmitted/founded viruses are expressed in transfected cells.
HEK293T cells were co-transfected with pCMV-IFITM3 (0.50 µg) and pCl-Nef encoding the indicate Nef protein (0.25 µg), and whole cell lysates were subjected to SDS-PAGE and immunoblotting with anti-Nef, anti-IFITM3, and anti-Actin (which served as loading control). Numbers and tick marks left of blots indicate position and size (in kilodaltons) of protein standard in ladder.

Supplemental File 1: The complete list of Nef proteins used in this study.

>NL4-3
MGGKWSKSSVIGWPAVRERMRRAEPAADGVGAVSRDLEKHGAITSSNTAANNAACA
WLEAQEEEEVGFPVTPQVPLRPMTYKAAVDLSHFLKEKGGLEGLIHSQRRQDILDLWIY
HTQGYFPDWQNYTPGPGVRYPLTFGWCYKLVPVEPDKVEEANKGENTSLLHPVSLHG
MDDPEREVLEWRFDSRLAFHHVARELHPEYFKNC

>SF2
MGGKWSKRSMGGWSAIRERMRRAEPRAEPAADGVGAVSRDLEKHGAITSSNTAATN
ADCAWLEAQEEEEVGFPVRPQVPLRPMTYKAALDISHFLKEKGGLEGLIWSQRRQEIL
DLWIYHTQGYFPDWQNYTPGPGIRYPLTFGWCFKLVPVEPEKVEEANEGENNSLLHP
MSLHGMEDAEKEVLVWRFDSKLAFHHMARELHPEYYKDC

>LAI
MGGKWSKSSVVGWPTVRERMRRAEPAADGVGAASRDLEKHGAITSSNTAATNAACA
WLEAQEEEEVGFPVTPQVPLRPMTYKAAVDLSHFLKEKGGLEGLIHSQRRQDILDLWIY
HTQGYFPDWQNYTPGPGVRYPLTFGWCYKLVPVEPDKVEEANKGENTSLLHPVSLHG
MDDPEREVLEWRFDSRLAFHHVARELHPEYFKNC

>97ZA012
MGGKWSKSSLVGWPNVRERMRRTEPAAEGVGAASRDLDKHGALTSSNTAHNNADCA
WLQAQEETEEVGFPVRPQVPLRPMTYKAAIDLSFFLKEKGGLEGLIHSKRRQDILDLWV
YHTQGYFPDWQNYTPGPGVRYPLTFGWCFKLVPVDPSEVEEANKGENNCLLHPMSQ
HGIEDAEREVLKWEFDSSLARRHIAREKHPEYYKDC

>NL43_97ZA012
MGGKWSKSSVIGWPAVRERMRRAEPAADGVGAVSRDLEKHGAITSSNTAANNAACA
WLEAQEEEEVGFPVTPQVPLRPMTYKAAVDLSHFLKEKGGLEGLIHSQRRQDILDLWIY
HTQGYFPDWQNYTPGPGVRYPLTFGWCFKLVPVDPSEVEEANKGENNCLLHPMSQH
GIEDAEREVLKWEFDSSLARRHIAREKHPEYYKDC

>97ZA012_NL43
MGGKWSKSSLVGWPNVRERMRRTEPAAEGVGAASRDLDKHGALTSSNTAHNNADCA
WLQAQEETEEVGFPVRPQVPLRPMTYKAAIDLSFFLKEKGGLEGLIHSKRRQDILDLWV
YHTQGYFPDWQNYTPGPGVRYPLTFGWCYKLVPVEPDKVEEANKGENTSLLHPVSLH
GMDDPEREVLEWRFDSRLAFHHVARELHPEYFKNC

>94UG114
MGGKWSKSSIVGWPAVRERMRRTEPAAEGVGAASRDLEKHGAITSSNTAQTNDACA
WLEAQEEEEVGFPVRPQVPLRPMTYKEAVDLSHFLKEKGGLEGLVWSPKRQEILDLW
VYHTQGFFPDWQNYTPGPGIRYPLTFGWCFELVPMEPKEVEENTEGEDNCLLHPINQ
HGMEDPEREVLVWRFNSRLAFEHKAKMKHPEYYKDC

>93BR020

MGGKWSKSSIVGWPAIRERMRRTPPTPPAAEGVGAVSQDLERRGAITSSNTRANNPD
LAWLEAQEEDEVGFPVRPQVPLRPMTYKGAVDLSHFLKEKGGLEGLIYSKRRQEILDL
WVYHTQGYFPDWQNYTPGPGIRYPLTMGWCFKLVPVDPEEVEKANEGENNCLLHPM
SQHGMEDEDKEVLKWEFDSRLALRHIARERHPEYYQD

>90CF056
MGGKWSKSRMGGWSTIRERMRRAEPVAEGVGAVSRDLDRRGAVTINNTASTNRDAA
WLEAQEDGEEVGFPVRPQVPLRPMTYKGAFDLSHFLKEKGGLDGLIYSKQRQDILDL
WVYNTQGYFPDWQNYTPGPGERFPLTFGWCFKLVPVNPQEVEQANEGENNSLLHPM
SLHGMEDDGREVLMWKFDSRLALTHLARVKHPEYKDC

>Acute_cladeC_Consensus
MGGKWSKSSIVGWPAVRERIRRTEPAAEGVGAASQDLDKHGALTSSNTAHNNADCAW
LQAQEEEEEVGFPVRPQVPLRPMTYKAAFDLSFFLKEKGGLDGLIYSKKRQEILDLWVY
HTQGFFPDWQNYTPGPGVRYPLTFGWCFKLVPVDPREVEEANKGENNCLLHPMSQH
GMEDEEREVLKWKFDSSLARRHLARELHPEYYKDC

>HIV-2_BEN
MGASGSKKLSKHSRGLRERLLRARGDGYGKQRDASGGEYSQFQEESGREQNSPSC
EGQQYQQGEYMNSPWRNPATERQKDLYRQQNMDDVDSDDDDLIGVPVTPRVPRRE
MTYKLAIDMSHFIKEKGGLQGMFYSRRRHRILDIYLEKEEGIIPDWQNYTHGPGVRYPM
YFGWLWKLVSVELSQEAEEDEANCLVHPAQTSRHDDEHGETLVWQFDSMLAYNYKAF
TLYPEEFGHKSGLPEKEWKAKLKARGIPYSE

>SIVmac239
MGGAISMRRSRPSGDLRQRLLRARGETYGRLLGEVEDGYSQSPGGLDKGLSSLSCE
GQKYNQGQYMNTPWRNPAEEREKLAYRKQNMDDIDEEDDDLVGVSVRPKVPLRTMS
YKLAIDMSHFIKEKGGLEGIYYSARRHRILDIYLEKEEGIIPDWQDYTSGPGIRYPKTFG
WLWKLVPVNVSDEAQEDEEHYLMHPAQTSQWDDPWGEVLAWKFDPTLAYTYEAYVR
YPEEFGSKSGLSEEEVRRRLTARGLLNMADKKETR

>CH040
MGGKWSKCSVVGWPSVRERMRRAEPAAEGVGAVSRDLEKHGAITSSNTAATNADCA
WLEAQEEGEVGFPVRPQVPLRPMTFKGALDLSHFLKEKGGLEGLIYSQKRQDILDLWV
YHTQGYFPDWQNYTPGPGTRFPLTFGWCFKLVPVDPGKVEEANKGENNCLLHPMSQ
HGMDDPEREVLVWRFDSSLAFRHVARELHPEYYKNC

>CH058
MGGKWSKRSVPGWADVRERMRRTEPRTEPAADGVGAVSRDLEKHGAITSSNTAANN
PDCAWLEAQEEEEEVGFPVRPQVPLRPMTYKGALDLSHFLKEKGGLEGLIHSQKRQDI
LDLWVYHTQGYFPDWQNYTPGPGTRYPLTFGWCFKLVPVDPEKVEEANTGENISLLH
PMSQHGMDDPEKEVLKWTFDSHLAFHHMARELYPEYYKN

>CH077
MGGKWSKFAGWPAVRERMRRAGARERRRRDEPAAVGVGPASQDLAKHGAITSSNTV
SNNADCAWLEAQEEEEEVGFPVRPQVPVRPMTYKAALDLSHFLKEKGGLEGLIYSQQ

RKDILDLWVYNTQGFFPDWQNYTPGPGPRFPLTFGWCFKLVPVEPEEVEKANEGENN
CLLHPMSQHGTDDPEKEVLAWRFDSRLAFQHVAREIHPEFYKDC

>SUMA
MGGKWSKSRGVGWSTIREKMRRAEPAAEPAAEGVGAVSRDLEKHGAITNSNTAATNA
DVAWLEAQEDEEVGFPVRPQVPLRPMTYKGAFDLSHFLKEKGGLEGLIYSRKRQEILD
LWVYHTQGYFPDWQNYTPGPGIRYPLTFGWCFKLVPVEPEEVEKANEGESNCLLHPM
SQHGMDDPEKEVLVWKFDSRLAFHHMARELHPEYYKDC

>TRJO
MGGKWSKRSVVGWPKVRERMRRVEPAADGVGAVSRDLDQRGAVTINNTPANNDTCA
WLEAQEDEDVGFPVRPQVPLRPMTFKGALDLSHFLKEQGGLDGLIYSQKRQEILDLWI
YHTQGYFPDWGNYTPGPGIRYPLTFGWCFKLVPVDPDEVEKANEGENNCLLHPMSQ
HGMDDPEKEVLMWKFDSMLAFQHKARELYPDYYKDC

>WITO
MGGKWSKSWKIGWPTVRERMRRAEPEPAAVGVGAVSRDLERHGAVTSSNTATNNAD
SAWLEAQAQEEDNEVGFPVRPQVPVRPMTYKAAVDLSHFLKEKGGLDGLIYSQQRQD
ILDLWVYNTQGFFPDWQNYTPGPGTRYPLTFGWCYKLVPVEPEEVEKANEGENNSLL
HPMGLHGMDDPEKEVLMWKFDSRLAFHHMAREKHPEFYKDC

Nef	Accession
NL4-3	AGL78171.1
SF2	P03407.3
LAI	P03406.3
97ZA012	AAK30998.1
94UG114	AAC97574.1
93BR020	AAA99884.1
90CF056	AF005496.1
HIV-2 Ben	M30502.1
SIVmac239	AAB99967.1
CH040	ACR51130.1
CH058	QZK27722.1
CH077	QZK27743.1
SUMA	ACR52828.1
TRJO	AAG34603.1
WITO	ACR52994.1
